# Single-molecule detection of oligonucleotides using the fluorescent nucleobase analogue ABN†

**DOI:** 10.1039/d4sc07334g

**Published:** 2025-02-03

**Authors:** George N. Samaan, Andres Jimenez Salinas, Alexandra E. Bailie, Julian Grim, Julian M. Cizmic, Anita C. Jones, Youngkwang Lee, Byron W. Purse

**Affiliations:** a Department of Chemistry and Biochemistry, San Diego State University San Diego CA USA youngkwang.lee@sdsu.edu bpurse@sdsu.edu; b The Smart Health Institute, San Diego State University San Diego CA USA; c School of Chemistry, The University of Edinburgh Edinburgh UK A.C.Jones@ed.ac.uk

## Abstract

Fluorescent nucleobase analogues (FBAs) have emerged as powerful tools for understanding nucleic acid systems at the molecular level. However, their application at the single-molecule level has been limited by low brightness and an incomplete understanding of how local chemical environments affect their properties. In this study, we investigate the bright fluorescent pyrimidine analogue ABN in duplex DNA oligonucleotides and study its single-molecule applications. Time-resolved fluorescence spectroscopy reveals its unique tautomeric behavior, including photo-induced double proton transfer, influenced by base-pairing partners. This tautomerization directly impacts ABN's quantum yield and spectral characteristics. By favoring a high quantum yield thymine-like tautomer through base pairing, surface-immobilized ABN-containing DNA duplexes are readily observed as bright spots using single-molecule fluorescence microscopy, exhibiting well-defined single-exponential bleaching kinetics. The brightness and photostability are enhanced by oxygen depletion. These results demonstrate that ABN is unique among FBAs in enabling single-molecule fluorescence studies of oligonucleotides using a standard microscopy setup.

## Abstract 2

We investigate the fluorescent pyrimidine analogue ABN in duplex DNA oligonucleotides, showing that ABN is unique among fluorescent nucleobase analogues in enabling single-molecule fluorescence studies of oligonucleotides using standard equipment.

## Graphical abstract

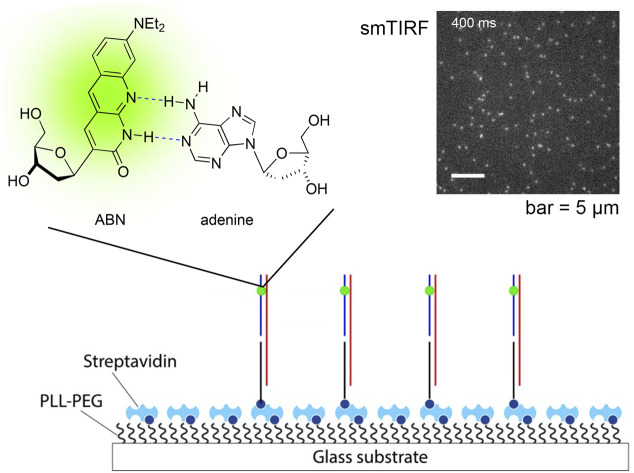

## Introduction

Single-molecule fluorescence studies can provide a detailed understanding of biomolecular function by allowing distinct conformations and biomolecular recognition events to be measured.^1–3^ Some of these conformations or molecular recognition complexes may be highly biologically significant but-little populated in the ensemble, making them very difficult or impossible to study in bulk measurements.^4^ Because of the low photon flux emitted by single fluorophores, high intensity irradiation and sensitive detection is needed. Fluorophores suitable for these conditions must be strongly absorbing and emitting, with a brightness of *ε*·*Φ*_em_ > 10 000 M^−1^ cm^−1^ preferred.^5,6^ Relatively long absorption and emission maxima, above 400 nm, minimize competing absorption by biological chromophores, and high photostability offers longer observation times.^7^ Most organic fluorophores that meet these criteria and are widely used in single-molecule fluorescence experiments are derivatives of a few privileged scaffolds, including Alexa fluors, rhodamines, and cyanines.^5,8^ For biophysical applications, these fluorophores are most often tethered to biomolecules using a flexible linker and common coupling reactions, such as amine–NHS ester coupling or thiol–maleimide conjugation. When applied to nucleic acids, these fluorophores are most often conjugated to functionalized termini, but specialized phosphoramidites can also be used during solid-phase oligonucleotide synthesis to provide reactive handles at internal positions to which fluorophores can be attached.^4^ Fluorophores tethered in this fashion to biological molecules—extrinsic labeling—have been used in many applications, but the tethers limit the precision of fluorophore placement and can interfere with the native behavior of biomolecules. An attractive alternative is to use intrinsically fluorescent derivatives of natural biomolecular building blocks, in this case nucleobases, but given the structural constraints of biomimicry, it has been challenging to obtain high brightness and photostability.^9–17^ To date, the best performing fluorescent nucleobase analogues (FBAs) that approach single-molecule detection in oligonucleotides are pA and MeO^th^aU, which have been detected using two-photon (2P) excitation at the level of 5 molecules and 7 molecules, respectively.^18,19^ Another bright FBA DMA^th^aU has been detected at the single-molecule level as a free nucleoside using multiphoton excitation, but the authors have reported difficulty with incorporating it into oligonucleotides.^19,20^ 2P excitation was used in these studies because pA, MeO^th^aU, and DMA^th^aU do not absorb significantly at wavelengths greater than 400 nm; 2P excitation at around 800 nm reduces background fluorescence and photobleaching, which facilitates detection, and is useful for live-cell imaging with fluorophores that are excited at shorter wavelengths.^21^ Work on other bright FBAs has been described in several recent reviews.^10,22,23^

**Fig. 1:**
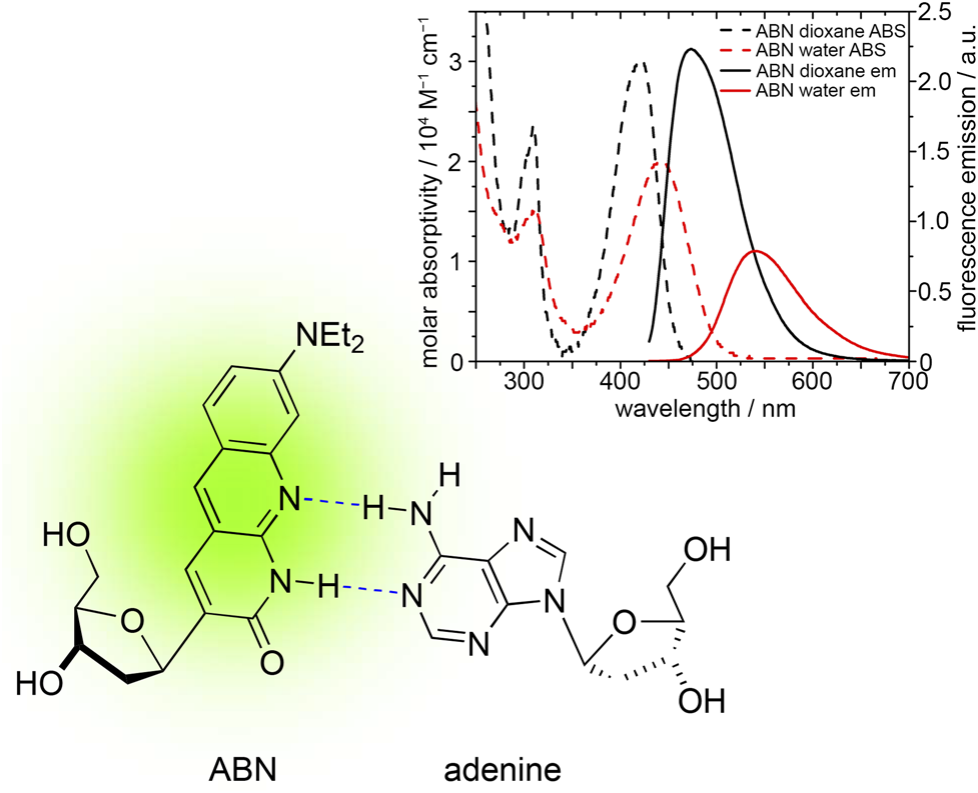
The structure of ABN in a base pair with adenine. The absorption (dashed) and emission (solid) spectra of ABN in 1,4-dioxane (black) and water (red) are inset.

To address this challenge, we designed and synthesized the fluorescent nucleoside analogue ABN, which exhibits *ε*_442 nm_ = 20 000 M^−1^ cm^−1^ and *Φ*_em_ = 0.39 in 1× PBS buffer at pH 7.4 (Fig. 1).^24^ The design of ABN was inspired by xanthene fluorophores, such as fluorescein and rhodamine B, which feature an electron-donating group in conjugation with an electron-withdrawing group (a “push–pull motif”) in a tricyclic framework. To incorporate these features into a fluorescent nucleoside analogue (FNA), we developed a *C*-linked ribonucleoside design wherein the carbonyl group at position 3 (xanthene numbering) plays the “pull” role of electron acceptor. In our first publication reporting on ABN, we showed that single molecules of the nucleoside immobilized on a glass surface are readily detected by single-molecule total-internal-reflection fluorescence (smTIRF) microscopy and fluorescence correlation spectroscopy.^24^ We found that ABN's high brightness is retained when base-paired and stacked in duplex nucleic acids. While the identity of neighboring bases has some influence on *Φ*_em_, this quantum yield was on average 20% greater in the duplex than for ABN as a free nucleoside.

In the present study, we sought to measure the performance of ABN in single-molecule detection of oligonucleotides using smTIRF and we performed time-resolved fluorescence measurements of ABN-containing oligos in the ensemble, aimed at understanding how tautomerism and base pairing influence its properties. An improved synthesis of ABN is also reported. Our results show that ABN is sensitive to quenching by molecular oxygen, but that excellent quality smTIRF data can be obtained under oxygen-depleted conditions. ABN is the first nucleobase analogue fluorophore that is readily detectable at the single-molecule level using conventional smTIRF instrumentation.

## Results and discussion

**Scheme 1:**
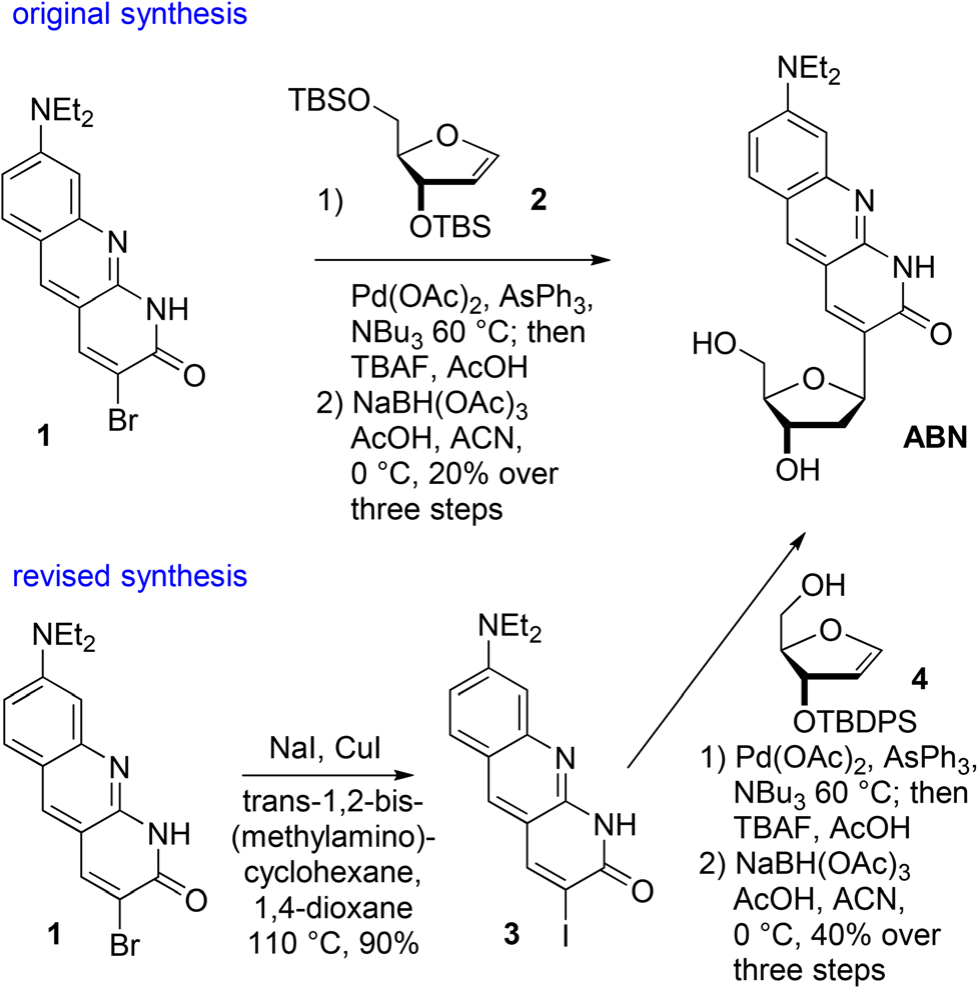
A revised synthesis of ABN uses a more reactive aryl iodide in a Heck reaction with a less sterically crowded, singly protected glycal 4, resulting in improved yield.

ABN was originally synthesized in a longest linear sequence of seven steps from the commercially available precursor 3-(diethylamino)acetanilide and the 3′,5′-bis(*O*-TBS) glycal 2.^24^ The key step, following synthesis of the ABN chromophore, is a Heck reaction to form a C–C bond in place of a C–N glycosidic bond (Scheme 1).^25–27^ While satisfactory for producing ABN, the reaction yields were lower and more variable than desired. The challenge was the Heck reaction of the aryl bromide of the ABN chromophore 1, whose electron richness would be expected to inhibit oxidative addition to Pd(0), typically the Heck reaction's rate-determining step. To improve the synthesis, we performed a Cu(i)-catalyzed transhalogenation of 1 to generate aryl iodide 3.^28^ We also substituted singly-protected glycal 4 for 3′,5′-bis(*O*-TBS) glycal 2, expecting that reduced steric crowding of the β-face and the potential of the 5′-OH to engage in productive coordination to Pd would be beneficial.^26^ These revisions improve the consistency of yield in the synthesis, delivering ABN in 36% yield over the last four steps, which compares favorably to a 20% yield over the last three steps in the original route (detail synthetic methods are presented in the ESI†). The 5′-*O*-DMTr-protected ABN phosphoramidite was prepared following the published method and the identity and purity were confirmed by ^1^H and ^31^P NMR spectroscopy.

**Fig. 2:**
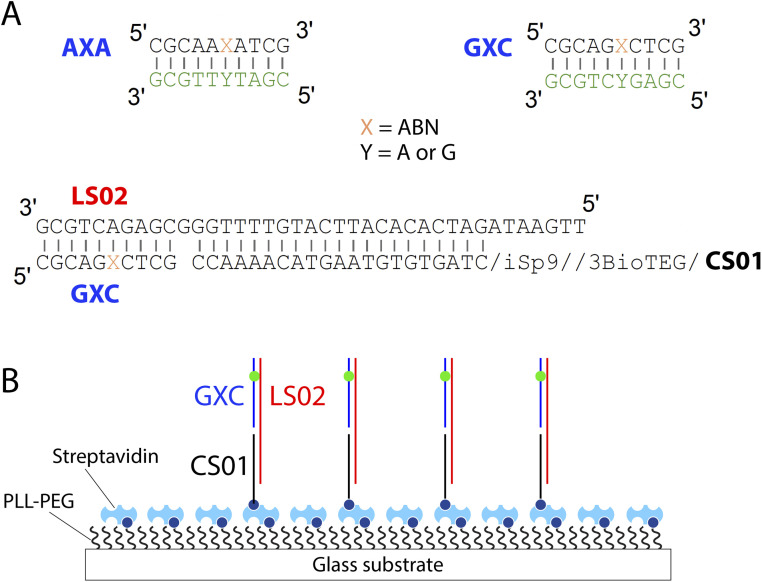
(A) Design of ABN-containing oligonucleotides AXA and GXC, complementary strands, biotinylated capture strand CS01, linker strand LS02, and (B) a cartoon of oligonucleotide immobilization on a PLL-PEG–streptavidin surface. iSp9 is a triethylene glycol spacer and 3BioTEG is a 3′ biotin with a triethylene glycol space (for complete structural descriptions, see ESI†).

To prepare for measurements of ABN's fluorescence properties at the single-molecule level in oligonucleotides, we selected 10-mer sequences that place ABN in two neighboring base contexts and a biotinylated capture strand design for surface immobilization (Fig. 2). These oligonucleotides were prepared by solid-phase DNA synthesis using standard conditions, with a 10-fold elongated coupling time for ABN (for details and additional sequences, see the ESI†). ABN was positioned between A and A (AXA) and between G and C (GXC). AXA and GXC match the sequences of the ABN-containing oligos ODN4 and ODN7 that we have studied previously; these neighboring base contexts were selected to match those used in past studies of other FBAs wherein the AXA and GXC local base stacking environments significantly impacted the FBAs.^16^ Changes in oligonucleotide sequence at positions not directly neighboring an FBA typically have little effect on fluorescence, except in specific cases wherein Förster resonance energy transfer (FRET) or photoinduced electron transfer (PET) operates between bases.^29–32^ We have not examined pyrimidine–pyrimidine “mispairs” with ABN in this study (*i.e.* ABN opposite C or T in a duplex) because the size mismatch of such pairs (as compared with a purine–pyrimidine base pair) makes them considerably perturbing to local structure.^33^ For surface immobilization and smTIRF microscopy, we chose a well-characterized immobilization architecture based on PLL-PEG–streptavidin adsorbed on a glass surface.^34^ The use of a common 3′-biotinylated capture strand CS01 and a tunable linker strand LS02, which is made up of only natural nucleotides, makes it easy to change the sequence of the linker and immobilize any desired ABN-containing oligonucleotide by hybridization.

To prepare for smTIRF of ABN in oligonucleotides, we first sought to better understand ABN's base pairing with purine partners. In our first publication on ABN, we found that ^13^C and ^1^H NMR of the free nucleoside in organic solvents supports the predominance of a thymidine-like tautomer (as shown in Fig. 1), a result that agrees with computational prediction of the tautomers' relative stability and matches what has been observed for simpler 1,8-naphthyridin-2(1*H*)-ones.^24^ Despite this finding, we observed no clear thermodynamic preference for base pairing with A over G, as indicated by the difference in melting temperature Δ*T*_m_ between ABN-containing *vs.* unmodified duplexes (Table 1).^24^ ABN is brightly fluorescent in all cases with the quantum yield *Φ*_em_ ranging from 0.29 to 0.55, depending on neighboring base context and base pairing. We have observed similar brightness of ABN in the single-stranded oligos AXA and GXC, where *Φ*_em_ = 0.49 and 0.62, respectively. We proposed that ABN has a T-like hydrogen bonding pattern when base-paired with A and forms either a wobble or a tautomeric base pair with G.

**Table 1:** Wavelengths of absorption and emission maxima, fluorescence quantum yields, and melting temperatures *T*_m_ for the ABN-containing duplex oligonucleotides. Quantum yields were measured at an excitation wavelength of 440 nm. Estimated uncertainty in quantum yield values is ±10%. Δ*T*_m_ = *T*_m_ for ABN-containing duplex listed in the table row – *T*_m_ for the corresponding duplex with canonical T in place of ABN

Oligo	*λ*_abs_/nm	*λ*_em_/nm	*ϕ*_em_	*T*_m_/°C	Δ*T*_m_/°C
AXA(A)	445	530	0.54	39.9 ± 0.3	−0.5
AXA(G)	470	521	0.17	41.3 ± 0.3	−7.3^a^
GXC(A)	450	538	0.48	34.3 ± 0.3	−14.1
GXC(G)	470	531	0.34	37.9 ± 0.3	−11.8^a^

a Comparison with *T*_m_ for the corresponding natural duplex with a C:G base pair.

**Fig. 3:**
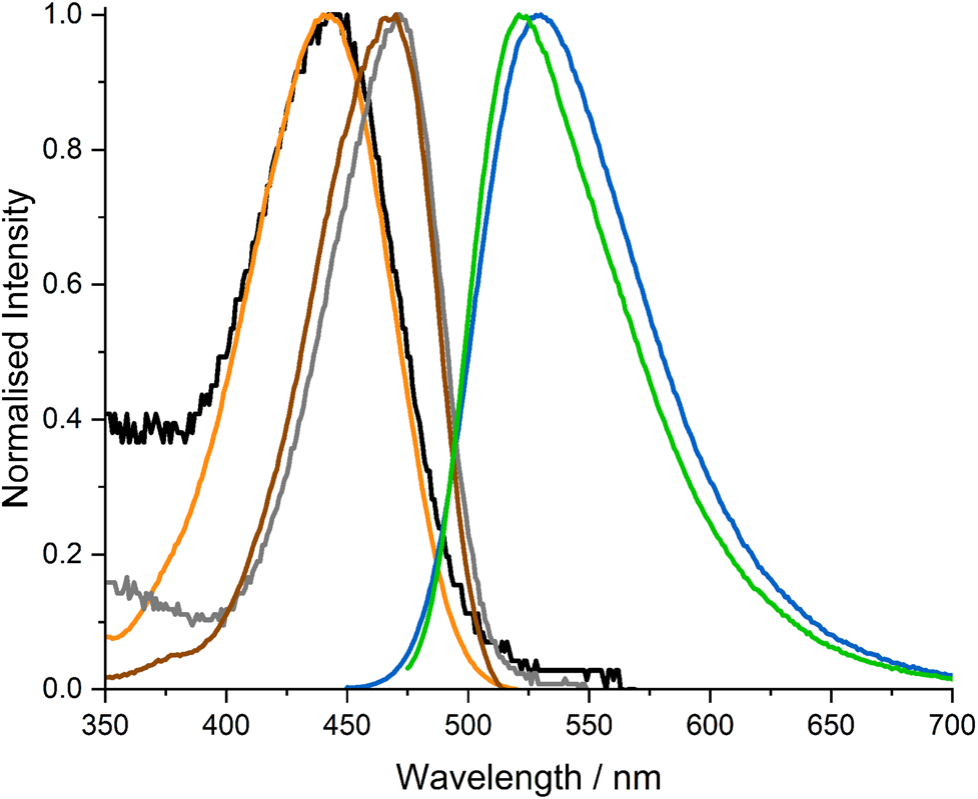
Normalised absorption, excitation and emission spectra of ABN in duplex oligonucleotides AXA(A) and AXA(G). Spectra of AXA(A): absorption (black), excitation (orange) (recorded at emission wavelength 526 nm), emission (blue) (recorded at excitation wavelength 445 nm). Spectra of AXA(G): absorption (grey), excitation (brown) (recorded at emission wavelength 520 nm), emission (green) (recorded at excitation wavelength 470 nm).

**Fig. 4:**
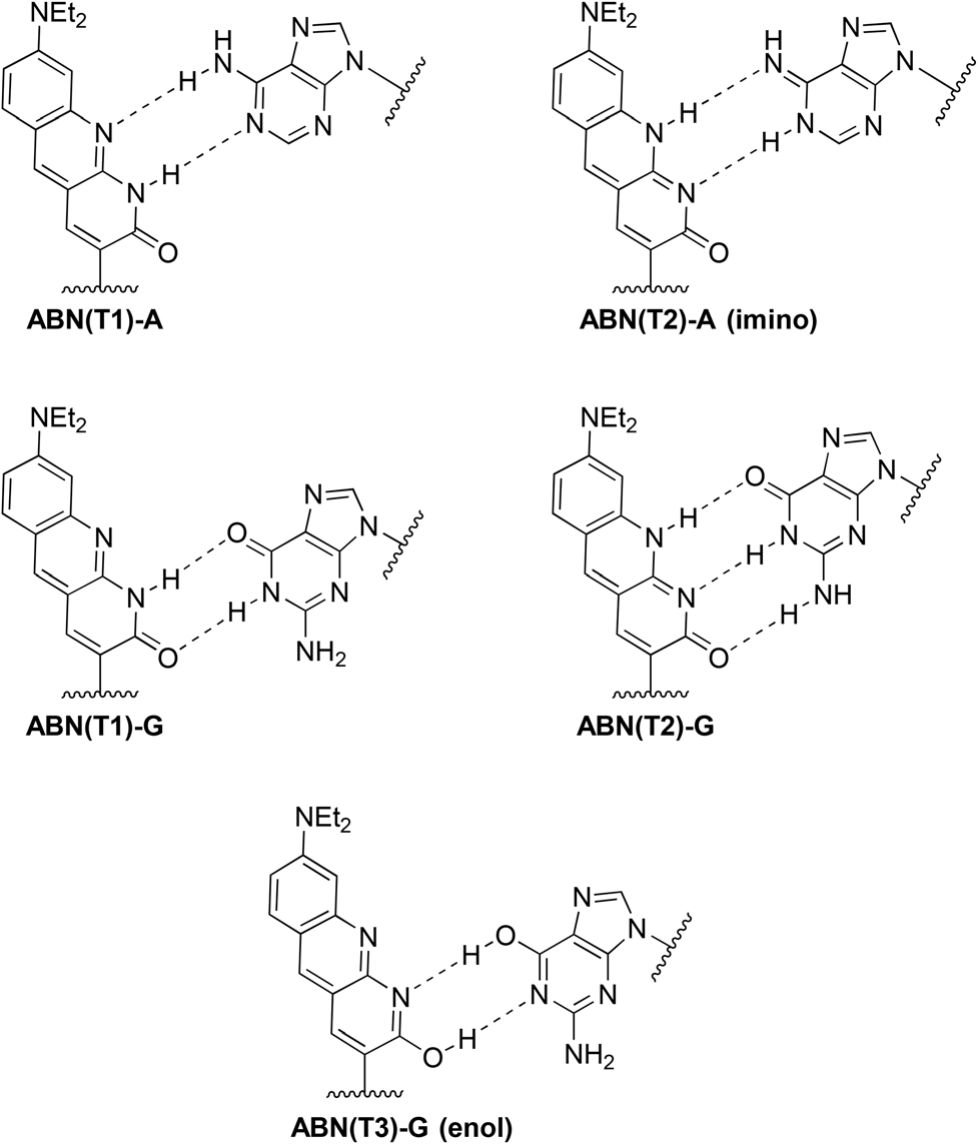
Base-pairing patterns of ground-state tautomers of ABN in DNA. ABN(T1)–A Watson–Crick base pair between T-like T1 and A; ABN(T2)–A(imino) formed from ABN(T1)–A by excited-state double proton transfer; ABN(T1)–G wobble base pair between T1 and G; ABN(T2)–G Watson–Crick base pair between C-like T2 and G; ABN(T3)–G (enol) formed from ABN(T1)–G by excited-state double proton transfer.

In the present study, we carried out a more detailed examination of ABN in duplex DNA oligonucleotides. Here we find that the absorption, excitation and emission spectra of ABN have little dependence on sequence context but are affected significantly by changing the base-pair partner of ABN from A to G (Fig. 2A). This finding is exemplified by the spectra of duplexes AXA(A) and AXA(G), where the nucleotide in parenthesis indicates the base-pair partner of ABN (Fig. 3; the spectra of the other duplexes are shown in Fig. S3 in ESI†). For AXA(A) the absorption and excitation spectra are nearly identical, but for AXA(G) there is a discernible discrepancy, suggesting a dependence of quantum yield on excitation wavelength (as confirmed by time-resolved fluorescence discussed below). For a single fluorescent species (*e.g.* one tautomer), the absorption and excitation spectra are identical because the quantum yield is independent of excitation wavelength. However, the presence of multiple species, which have different excitation spectra and different quantum yields, results in a dependence of the average quantum yield on excitation wavelength. Changing the base-pair partner from A to G causes a significant bathochromic shift in the absorption spectrum, which can be related to the existence of ABN in different ground-state tautomeric forms in the two duplexes. ABN exists as a thymine-like tautomer when base-paired with A and tautomerises to adopt a cytosine-like hydrogen bonding pattern to support base pairing with G (Fig. 4). Further evidence for this tautomeric change is provided by the time-resolved fluorescence studies discussed below. The fluorescence quantum yield depends on both sequence context and base-pair partner, but is more greatly influenced by the latter, with a significant decrease on going from A to G (Table 1).

**Fig. 5:**
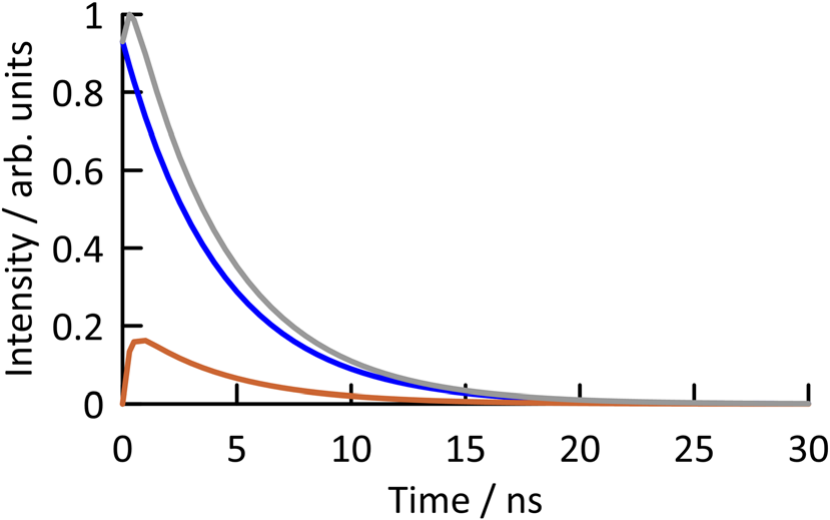
The function fitted to the observed fluorescence response function of ABN in 1,4-dioxane, excited at 440 nm and detected at 505 nm, is shown in grey (see Fig. S4 for experimental data†). It consists of the sum of the response functions of T1 (blue) and T2 (orange). The emission of T1 (directly excited) shows a mono-exponential decay with a lifetime of 4.2 ns. The emission of T2 (populated by excited-state tautomerization of T1) shows a rise time of 0.25 ns and a decay time of 4.2 ns.

To better understand how base pairing, stacking, and tautomerism govern ABN's fluorescent properties, we studied its time-resolved fluorescence responses, starting with characterizing ABN as a free nucleoside. Time-resolved fluorescence has been used to characterize other FBAs, providing a useful way to relate their structure to their fluorescent properties and responsiveness to changes in their local chemical environment, including base pairing.^18,20,35,36^ The time-resolved fluorescence response of ABN nucleoside in Tris buffer or 1,4-dioxane depends on the emission wavelength (Table 2). At shorter emission wavelength, a simple mono-exponential decay is observed with a lifetime of 4.2 ns in dioxane and 2.7 ns in buffer. We assign this decay to the thymine-like T1 tautomer of ABN, which is consistent with the NMR data and computational prediction that this tautomer is intrinsically more stable (Scheme 1).^24^ At long emission wavelength, an additional component is observed with a rise-time (Fig. 5, S4 and S5†). The observation of a rise-time in time-resolved fluorescence indicates indirect excitation of the emitting species *via* optical excitation of a different ground-state species. In the present case, the initially excited T1 tautomer undergoes excited-state intramolecular proton transfer to form a second tautomer, T2, which emits with a much shorter lifetime (see ESI† for details of the kinetics of excited-state tautomerization). We assign T2 as the cytosine-like tautomer (Fig. 4); this is confirmed by the measurements on ABN-containing oligonucleotides discussed below. Although T2 emits at slightly longer wavelength than T1, its emission overlaps that of T1 and could not be spectrally isolated. The observed fluorescence response consists of contributions from directly excited T1 and indirectly excited T2 (eqn (S4)†), as shown in Fig. 5 and S5.† The overlap of the emission spectra of T1 and T2 means that the phenomenon of dual fluorescence, that is often characteristic of excited-state proton transfer,^37^ is not obvious in ABN. We can infer that, in both solvents, ABN exists as T1 in the ground state, but can be photo-tautomerised to form T2 in the excited state. The fluorescence lifetimes of T1 and T2 are solvent-dependent, both decreasing on going from 1,4-dioxane to Tris, from 4.2 ns to 2.7 ns for T1 and from 0.25 to 0.12 ns for T2 (Table 2). This is consistent with the previously reported decrease in quantum yield from 0.64 to 0.39.^24^ The steady-state fluorescence spectrum of ABN in both solvents is dominated by T1 (>80% of the intensity).

**Table 2:** Fluorescence lifetimes of the tautomers of ABN for the free nucleoside in solution and for ABN in the duplex oligonucleotides AXA(A) and AXA(G), and their respective % contributions to the steady-state intensity at the emission wavelengths shown; * indicates that this lifetime component was observed as a rise-time. Emission was excited at 440 nm

Sample	*λ*_em_/nm	*τ*_1_/ns	*τ*_2_/ns	% SS_T1_	% SS_T2_
ABN in dioxane	475	4.2	—	100	
	505	4.2	0.25*	82	18
ABN in Tris	510^a^	2.7	—	86	—
	570	2.7	0.12*	89	11
ABN in duplex AXA(A)^b^	500^a^	7.2	1.8*	78	21
	530			77	23
	560			75	25
ABN in duplex AXA(G)^b^	490^a^	7.0	1.4	33	61
	520			45	55
	550			63	37

a A minor additional decay component was present at this emission wavelength (see ESI Tables S1 to S3).

b Lifetimes were obtained by global analysis of response functions recorded at three excitation and three emission wavelengths (see ESI Tables S4 and S5).

**Fig. 6:**
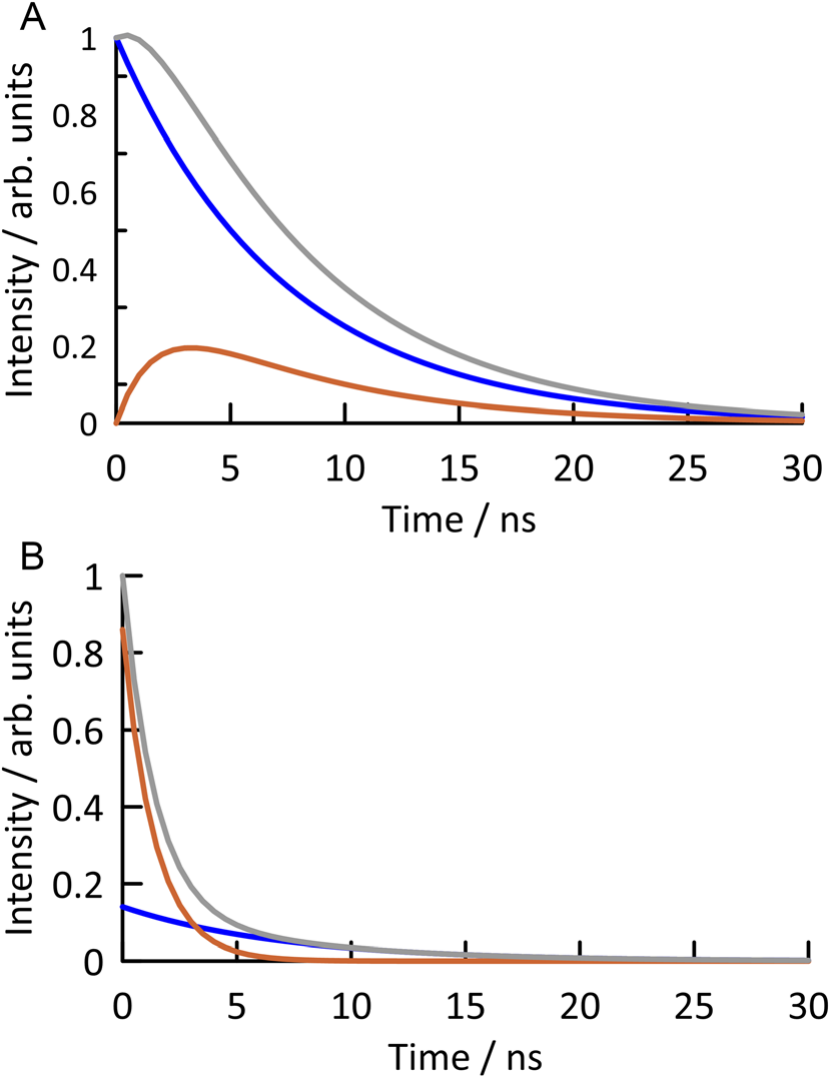
(A) The function fitted to the observed fluorescence response function of ABN in duplex oligonucleotide AXA(A), excited at 440 nm and detected at 530 nm, is shown in grey (see Fig. S8 for experimental data†). It consists of the sum of the response functions of T1 (blue) and T2 (orange). The emission of T1 (directly excited) shows a mono-exponential decay with a lifetime of 7.2 ns. The emission of T2 (populated by excited-state tautomerisation of T1) shows a rise time of 1.8 ns and a decay time of 7.2 ns. (B) The function fitted to the observed fluorescence response function of ABN in duplex oligonucleotide AXA(G), excited at 440 nm and detected at 520 nm, is shown in grey (see Fig. S9 for experimental data†). It consists of the sum of the mono-exponential decays of T1 (blue) and T2 (orange), with lifetimes of 7.0 ns and 1.4 ns, respectively.

The time-resolved fluorescence responses of ABN in duplexes further support the model of ground-state tautomerism to enable base pairing with G and explain spectral responses to base pairing. A rise-time is observed when ABN is paired with A, but not when it is paired with G, as illustrated by the response functions of AXA(A) and AXA(G) shown in Fig. 6 (the GXC duplexes show analogous behavior, as shown in Fig. S6 and S7; see the ESI†). For AXA(A) and AXA(G), the response functions were measured at 3 emission wavelengths for each of 3 excitation wavelengths. For the GXC duplexes, only one excitation wavelength was used.

For the ABN–A duplexes (AXA(A) and GXC(A)), the form of the fluorescence response is identical to that seen for the nucleoside at long emission wavelength (eqn (S4)†), the sum of the response functions of directly excited T1 and indirectly excited T2. Duplex AXA(A) shows a decay time of 7.2 ns (T1 lifetime) and a rise-time of 1.8 ns (T2 lifetime) at all excitation and emission wavelengths (Tables 3 and S4†). In this duplex, ABN is expected to exist as the thymine-like tautomer, enabling formation of a Watson–Crick base pair with A in the complementary strand (Fig. 4). T1 is thus confirmed as the thymine-like tautomer. In the duplex, indirect excitation of the C-like T2 involves excited-state double proton transfer (ESDPT) between T-like ABN and based-paired A to form a tautomeric base pair, with ABN in the C-like form and A in the imino form, as shown in Fig. 4. To our knowledge, this is the first experimental evidence to support ESDPT between a FBA and a natural base in DNA; the process has been predicted computationally to be energetically feasible for the pyrrolocytosine–guanine base pair.^38^ The steady-state emission intensity is dominated by T1 (∼80%) over the range 500–560 nm, with negligible dependence on emission wavelength. There is no significant dependence of the steady-state contributions on excitation wavelength (Table S4†), consistent with both emitting species being excited by the same absorption process.

**Table 3:** Fluorescence lifetimes (*τ*_i_) of the tautomers of ABN in the three duplex oligonucleotides studied; * indicates that this lifetime component was observed as a rise-time. The respective % contributions to the steady-state intensity and the number-average emission lifetime 〈*τ*〉 are also given, for excitation at 440 nm and detection of emission at *λ*_max_

Oligo	*τ*_1_/ns	*τ*_2_/ns	% SS_T1_	% SS_T2_	〈*τ*〉/ns
AXA(A)	7.2	1.8*	77	23	4.2
AXA(G)	7.0	1.4	45	55	2.2
GXC(A)	7.3	1.4*	87	13	4.8
GXC(G)^a^	7.0	3.0	56	38	3.6

a A minor additional decay component was present.

Significant differences are observed when ABN is base-paired with G. The ABN–G duplexes (AXA(G) and GXC(G)) show normal decay curves, with no rise-time and two decay times, identifiable with T1 and T2, at all excitation and emission wavelengths (Tables 2 and S5†). These data indicate the existence of both*tautomers* in the ground state. In the ABN–G duplexes, the existence of the C-like T2 in the ground state is now favored by its ability to form a Watson–Crick base pair with G (Fig. 4); this tautomer dominates the emitting population, with a fractional amplitude (*A*_2_) of around 0.8 at 520 nm. However, the T-like T1 tautomer remains populated as the minor species because it can form a wobble base-pair with G (Fig. 4). Now that T2 is populated in the ground state, it can be excited directly. An increase in its fractional contribution to the emitting population with increasing excitation wavelength can be seen (Table S5†), indicating that its absorption spectrum is red-shifted relative to T1. The red-shifted absorption spectrum of AXA(G) is thus accounted for by the population of the T2 tautomer in the ground state when ABN is base-paired with G (Fig. 3). The contribution of T2 to the steady-state emission intensity is greater than for AXA(A) and decreases with increasing emission wavelength (Table 2); this is consistent with the blue-shifted emission spectrum of AXA(G) (Fig. 3). The concentration dependent red-shift in the absorption spectra reported previously for the GXC single strand and the GXC(G) duplex also aligns with increase in the population of the C-like T2 tautomer in the ground state when ABN is paired with G (see Fig. S10 for further discussion†).^24^ The wavelength-dependent contribution of T2 to the emitting population results in dependence of the average lifetime, hence the quantum yield, on both emission and excitation wavelengths (Table S6†).

The lifetimes of T1 and T2 in all the duplexes are summarized in Table 3. The fluorescence lifetimes of both tautomers in the base-paired state are significantly longer than for the free nucleoside (Table 2), with the lifetime of T2 increased by an order of magnitude. The lifetime of T1 is essentially independent of sequence context, implying an absence of inter-base quenching interactions, such as charge transfer. It is also independent of base-pair partner, which suggests that ESDPT may be occurring in T1-G base pairs, but is not evident as a rise in the fluorescence response because it is masked by the much greater contribution from directly excited T2. The lifetime of T2 appears to be much more sensitive to molecular environment and is influenced by both base-stacking and pairing interactions. The number-average lifetime of ABN–G is consistently shorter than for ABN–A in the same sequence context, due to the greater contribution of the shorter-lived T2 to the emitting population. The average lifetimes show good correlation with the steady-state quantum yields (Tables 1 and 3), considering that the average lifetime, measured over a narrow emission band, is wavelength-dependent, and confirm the observed trends. Some minor features of the fluorescence response functions may be related to a small population of a third, enol tautomer (see Fig. 3 for the structure).

Having characterized how ABN's base pairing influences its fluorescence, we next studied the capabilities of ABN integrated into a DNA duplex as a single-molecule probe. Here we employed TIRF microscopy, which is widely used for studies of the structure and dynamics of nucleic acids and their associated proteins.^39^ We selected GXC for its high brightness and immobilized it by hybridizing with linker strand LS02, which is complementary to GXC at the 3′-end and to the biotinylated capture strand CS01 at the 5′ end (Fig. 2). In this configuration, ABN is paired with A and exhibits a reasonably high quantum yield of 0.48, which includes emission of the directly populated T1 tautomer and a minor component of emission of the T2 tautomer, which results from ESPT (Table 3 and Fig. S6†). GXC was selected over AXA because it shows a marginal, but practical, redshift in the excitation spectrum, better matching the excitation wavelength of the 488 nm laser. Emission light was collected over the range of 500–570 nm, encompassing both T1 and T2 emissions.

**Fig. 7:**
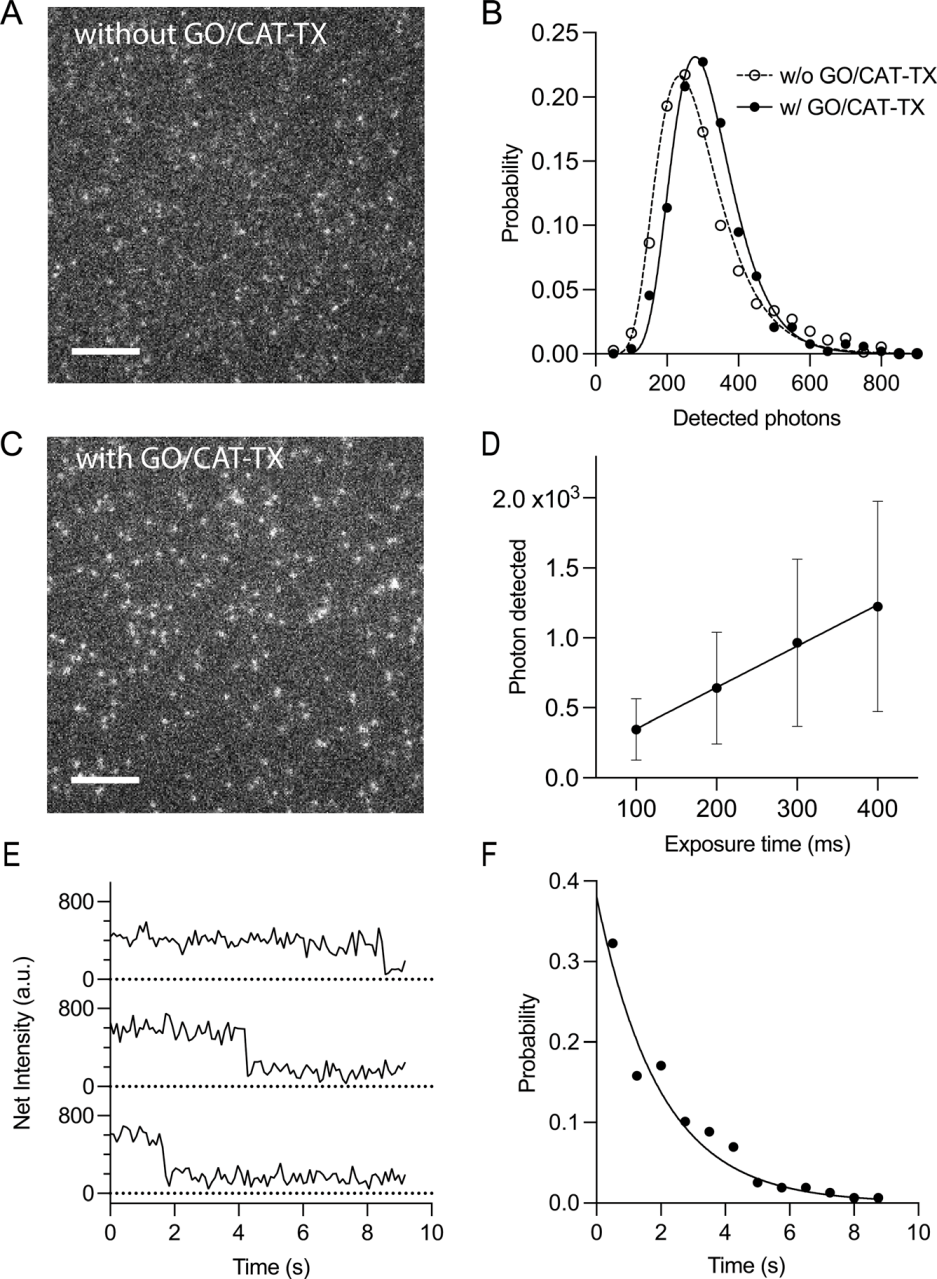
Single-molecule characterization of ABN incorporated in GXC. TIRF microscopy image of ABN in PBS (A) without and (C) with the addition of the oxygen scavenger GO/CAT-TX. Scale bars represent 5 μm. (B) A histogram showing the number of detected photons at an exposure time of 100 ms. The mean photon number *μ*, was estimated to be 289 ± 187 (*N* = 744) and 318 ± 202 (*N* = 524) with and without GO/CAT-TX, respectively. (D) A plot of number of detected photons as a function of exposure time. The number of photons exhibits a linear increase with exposure time, resulting in a brightness of 2.96 kHz per molecule. *N*_100ms_ = 300; *N*_200ms_ = 320; *N*_300ms_ = 276; *N*_400ms_ = 274. (E) Representative fluorescence intensity time traces of single-molecule ABN. Time resolution: 100 ms. (F) A plot of photobleaching/blinking kinetics of ABN in the presence of oxygen scavenging buffer. *k* = 0.50 s^−1^. *N* = 164.

We observed that spatially isolated individual molecules of ABN within a DNA duplex could be readily visualized with a short exposure time (100 ms) and at a moderate power density (0.07 kW cm^−2^; Fig. 7A). The mean number of detected photons *μ*, per individual molecule was estimated to be 289 ± 187. The fluorescence intensity follows a lognormal distribution (Fig. 7B), which is consistent with the distribution observed for ABN in its free nucleoside form.^24^ The high selectivity of immobilization was confirmed in a control experiment wherein streptavidin was omitted, resulting in the loss of signal (Fig. S11†). During our investigation into the photostability of ABN, we noticed that a significant portion of ABN experienced rapid photobleaching within 100 ms (see Fig. S12A†). The photostability of dyes is greatly impacted by the concentration of molecular oxygen.^40^ Quenching of the triplet states of dyes by molecular oxygen results in the formation of a higher energy singlet oxygen species.^41^ Subsequently, singlet O_2_ can rapidly react with chemical groups present in dyes, causing irreversible damage to them. We further investigated whether the elimination of molecular oxygen could improve the photostability of ABN. We tested both the protocatechuic acid/*Pseudomonas putida* protocatechuate-3,4-dioxygenase (PCA/PCD) and glucose-coupled glucose oxidase/catalase (GO/CAT) oxygen scavenging systems, which are widely used to scavenge oxygen in single-molecule fluorescence experiments.^42,43^ Trolox (TX) was supplemented as it is known for rapidly depopulating triplet states that accumulate in the absence of molecular oxygen.^44^ In our trials, the GO/CAT system provided 40% better resistance to photodarkening as compared with the PCA/PCD. In the presence of GO/CAT-TX, the brightness of ABN increased by 10% (*μ* = 318 ± 202) while maintaining a lognormal intensity distribution (Fig. 7B and C). Importantly, the photostability is greatly improved. The number of detected photons linearly increased with exposure time, yielding a brightness of 2.96 kHz per molecule, defined as the number of detected photons per second (Fig. 7D and S13†). Subsequently, we analyzed the photodarkening kinetics associated with blinking or bleaching with a resolution of 100 ms. Intensity traces of individual ABN-containing duplexes show clear single-step photobleaching and some of these duplexes remain bright for up to nine seconds (Fig. 7E). The fluorescence on-time was adequately fitted to a single-exponential distribution. The dissociation kinetics of the 10-mer ABN GXC(A) were sufficiently slow, with an estimated rate of 0.010 s^−1^, to have a minimal impact on the experimental results (Fig. S14†). When accounting for the dissociation kinetics, the photodarkening rate constant is estimated to be *k* = 0.50 s^−1^ (Fig. 7F; the corresponding photodarkening rate constant for Alexa488 is *k* = 0.13 s^−1^; see Fig. S15†), demonstrating that ABN has suitable kinetic properties for single-molecule study.

We compared the single-molecule detection of ABN incorporated into a DNA duplex with Alexa488 covalently attached to the 5′ end of an oligonucleotide (Fig. S15 and Table S7†). Alexa488 exhibited approximately four times greater brightness compared to ABN. The relatively weak brightness of ABN can be partially attributed to inefficient excitation with a 488 nm laser, as its peak excitation wavelength is at 450 nm; the molar absorption coefficient at 450 nm is approximately twice that at 488 nm (Fig. S3†). ABN is more sensitive to photodarkening than Alexa488, with a rate constant of 0.50 s^−1^, compared to 0.13 s^−1^ for Alexa488. While ABN does not fully match the brightness and photostability of widely used fluorophores for smTIRF such as Alexa488, its capabilities as an FBA for single-molecule detection of oligonucleotides are currently the best available.

## Conclusions

In this study, we sought to understand how ABN's base pairing and tautomerism influence its fluorescence properties and to measure its performance as an FBA capable of single-molecule applications. The time-resolved fluorescence spectroscopy study revealed that ABN predominantly adopts the thymine-like T1 tautomeric form as a free nucleoside, but part of its fluorescent response derives from intramolecular excited-state proton transfer to generate the cytosine-like T2 tautomer, which contributes to emission with a shorter lifetime. When base-paired with A, ABN primarily exists in the T1 tautomeric form, forming Watson–Crick-like base pairs. Approximately 80% of its emission results from an excited state that retains this configuration. A minor fraction (∼20%) of its emission originates from excited-state double proton transfer to form a base pairing between ABN in a cytosine-like donor–acceptor–acceptor configuration T2 and the imino tautomer of adenine. When ABN is base-paired with G, it exists predominantly as the C-like T2 tautomer and forms Watson–Crick-like base pairs. A minor fraction remains in the T-like tautomer, forming a wobble base pair. In this context, both T2 and T1 are directly excited and contribute to the fluorescence response. These differences in tautomeric populations between ABN:A and ABN:G base-pairs are manifest in characteristic differences in absorption and emission wavelengths, time-resolved fluorescence response functions and quantum yield. These base pair-dependent differences in fluorescence response will enable future users of this fluorophore to tune its optical properties as desired by controlling base pairing, and there is an exciting potential to use these differences to probe base pairing in structural and dynamic studies on nucleic acids.

The high brightness of ABN and its absorption and emission at relatively long wavelengths, compared with other FBAs, is especially enabling for single-molecule fluorescence. Individual ABN oligonucleotides in duplex DNA are readily detected using standard smTIRF instrumentation and the widely available 488 nm laser for excitation at moderate power density. Oxygen scavenging using GO/CAT and triplet depopulation using Trolox improves brightness and inhibits photobleaching, allowing time traces up to nine seconds with clear observation of single-step photobleaching. These results show that ABN is the first FBA to enable single-molecule fluorescence studies on oligonucleotides using widely available instrumentation and standard conditions for the study of biological molecules. We anticipate that the ability to translate the long-established advantages of FBA-labelling from ensemble to single-molecule studies will be transformative in advancing the understanding of nucleic acid structure and dynamics, in applications such as the study of protein–nucleic acid interactions or investigation of the individual structures that populate the RNA ensemble.

## Data availability

The data supporting this article have been included as part of the ESI.†

## Author contributions

George Samaan: conceptualization, investigation, methodology, resources. Andres Jimenez Salinas: investigation, methodology, visualization, formal analysis, and resources. Alexandra Bailie: investigation, formal analysis, visualization. Julian Grim: investigation. Julian Cizmic: investigation. Anita Jones: conceptualization, data curation, formal analysis, funding acquisition, resources, supervision, validation, visualization, writing – original draft preparation, writing – review & editing. Youngkwang Lee: conceptualization, data curation, formal analysis, funding acquisition, resources, supervision, validation, visualization, writing – original draft preparation, writing – review & editing. Byron Purse: conceptualization, data curation, formal analysis, funding acquisition, project administration, resources, supervision, validation, visualization, writing – original draft preparation, writing – review & editing. All authors have read and agreed to the published version of the manuscript.

## Conflicts of interest

There are no conflicts to declare.

## Supplementary Material

SC-OLF-D4SC07334G-s001
