# Supplementary material for: Single-molecule detection of oligonucleotides using the fluorescent nucleobase analogue ABN
Source: Chem Sci. 2025 Feb 3;16(11):4866–75. doi: 10.1039/d4sc07334g (PMC11808398; doi:10.1039/d4sc07334g)
Supplement: SC-016-D4SC07334G-s001 [file SC-016-D4SC07334G-s001.pdf]

Supplementary Information (SI) for Chemical Science.
This journal is © The Royal Society of Chemistry 2025

SUPPLEMENTARY INFORMATION

Single-molecule detection of oligonucleotides using the fluorescent nucleobase analogue ABN

George N. Samaan,[1,a] Andres Jimenez Salinas,[1,a] Alexandra E. Bailie,[3,a] Julian Grim,[1] Julian M. Cizmic,[1] Anita C. Jones,[3]* Youngkwang Lee,[1,2]* and Byron W. Purse[1]*

[1] Department of Chemistry and Biochemistry, San Diego State University, San Diego, CA, USA

[2] The Smart Health Institute, San Diego State University, San Diego, CA, USA

[3] School of Chemistry, The University of Edinburgh, Edinburgh, UK

* Correspondence: A.C.Jones@ed.ac.uk; youngkwang.lee@sdsu.edu; bpurse@sdsu.edu;

[a] G.N.S., A.J.S. and A.E.B. contributed equally to this work.

Table of Contents

1. Materials and Methods 2
  1.1 General Experimental 2
  1.2 Revised ABN Synthesis 3
  1.3 Synthesis of oligonucleotides 8
  1.4 Time-resolved fluorescence spectroscopy 9
  1.5. Single-molecule total internal reflection fluorescence (smTIRF) microscopy 12
2. Additional spectra, time-resolved fluorescence responses, and data tables for ABN in oligonucleotides 14
3. Supplementary figures and table for smTIRF 22
4. References 25

1. Materials and Methods

1.1 General Experimental

Starting materials were obtained in ACS reagent grade or higher from Acros Organics, Alfa Aesar, Fisher Scientific, Sigma-Aldrich and Wako Chemicals and used without further purification. Analytical thin-layer chromatography was performed on pre-coated 200 μm silica gel F-254 plates. Visualization was performed by ultraviolet light. Flash column chromatography was performed using a Teledyne-Isco CombiFlash RF 200 using UV/vis detection. ^{1}H NMR spectra were recorded on 400 and 500 MHz Varian spectrometers using an AutoX PFG probe at 298 K; residual solvent peaks were used as internal references: DMSO (quint, δH = 2.50 ppm), $CHCl_3$ (s, δH = 7.26 ppm) or methanol (quint, δH = 3.31 ppm). ^{13}C NMR spectra were recorded on 400 and 500 MHz Varian spectrometers using an AutoX PFG probe at 298 K; δ relative to DMSO (δ 40.50 ppm), $CHCl_3$ (δ 77.23 ppm) or methanol (δ 49.00 ppm). Coupling constants (*J*) are reported in hertz (Hz). The following abbreviations are used to describe the multiplicities: s = singlet, d = doublet, t = triplet, q = quartet, quint = quintet, sext = sextet, m = multiplet, dd = doublet-doublet, ddd = doublet-doublet-doublet, dt = doublet-triplet, dq =doublet-quartet. High-resolution electrospray ionization (ESI) mass spectrometry was performed using an Agilent 6530 Accurate-Mass Q-TOF LC/MS.

1.2 Revised ABN Synthesis

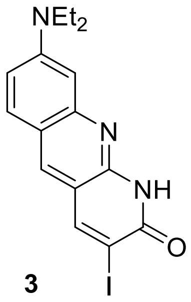

3

8-(diethylamino)-3-iodobenzo[*b*][1,8]naphthyridin-2(1*H*)-one (3).

In a sealed pressure tube, 8-(diethylamino)-3-bromobenzo[b][1,8]naphthyridin-2(1H)-one **1** (150 mg, 0.434 mmol; synthesized according to our previously reported method[1]), sodium iodide (220 mg, 1.47 mmol), copper I iodide (15 mg, 0.0789 mmol) and trans-N,N′-dimethylcyclohexane-1,2-diamine (25 µL, 0.158 mmol) were added, then dissolve in 4.50 mL of dioxane. The reaction was run at 110 °C for 18 h and monitored by crude NMR in $CDCl_3$. After reaction was done, water was added and the product extracted with DCM three times. The organic solvent was dried with anhydrous sodium sulfate then evaporated to give the product **3**, which was used in the following step with no further purification.

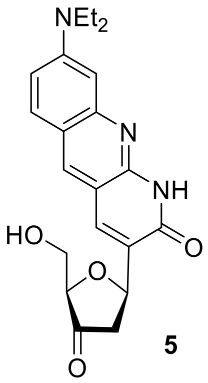

5

8-(diethylamino)-3-((2*R*,5*R*)-5-(hydroxymethyl)-4-oxotetrahydrofuran-2-yl)benzo[*b*][1,8]naphthyridin-2(1*H*)-one (5).

Palladium acetate (156 mg, 0.696 mmol) and triphenylarsine (426 mg, 1.39 mmol) are dissolved in 7.00 mL DMF and stirred for 30 min at room temperature. The reaction mixture is then transferred to another flask containing the crude product (**3**) (342 mg, 0.870 mmol) and 1,4-anhydro-3-*O*-(tert-butyldiphenylsilyl)2-deoxy-D-erythro-pent-1-enitol (**13**) (354 mg, 1.00 mmol), then tributylamine (413 µL, 1.74 mmol) is added. The reaction is heated to 60.0 °C and monitored by TLC (50% DCM in EtOAc). After 18 h, the reaction is cooled to room temperature then 0.500 mL acetic acid and 1M tetrabutylammonium fluoride (1.74 mL, 1.74 mmol) are added and the reaction is stirred for 1 h at room temperature. The solvent is then evaporated under vacuum and automated flash chromatography is performed (0–10% MeOH in DCM), with semi-pure product eluting at 5% MeOH in DCM.

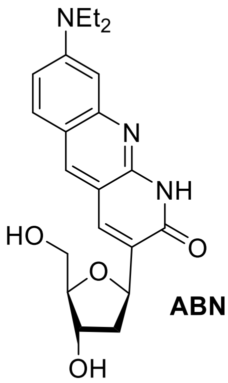

ABN

8-(diethylamino)-3-((2*R*,4*R*,5*R*)-4-hydroxy-5-(hydroxymethyl)tetrahydrofuran-2-yl)benzo[*b*][1,8]naphthyridin-2(1*H*)-one (ABN).

The semi-pure product (**5**) and sodium triacetoxyborohydride (277 mg, 1.31 mmol) are dissolved in 3.00 mL acetonitrile and 3.00 mL acetic acid at 0 °C. The reaction is stirred for 2 h at that temperature and monitored by TLC (10% methanol in DCM; R_f = 0.435). The solvent is then evaporated and automated flash chromatography is performed with the product eluting at 10% methanol in DCM. The solvent is evaporated to give 94.8 mg (40% yield over the last 3 steps) of yellow solid **ABN**.

^{1}H NMR (500 MHz, CD_3OD) δ 8.29 (s, 1H), 8.07 (d, *J* = 1.1 Hz, 1H), 7.74 (d, *J* = 9.2 Hz, 1H), 7.16 (dd, *J* = 9.3, 2.5 Hz, 1H), 6.88 (d, *J* = 2.4 Hz, 1H), 5.24 (dd, *J* = 10.0, 5.9 Hz, 1H), 4.33 (dt, *J* = 5.5, 2.4 Hz, 1H), 3.99 (td, *J* = 4.6, 2.9 Hz, 1H), 3.73 (qd, *J* = 11.8, 4.6 Hz, 2H), 3.56 (q, *J* = 7.1 Hz, 4H), 2.43 (ddd, *J* = 13.1, 5.9, 2.1 Hz, 1H), 2.00 – 1.93 (m, 1H), 1.26 (t, *J* = 7.1 Hz, 6H). **^{13}C NMR** (125 MHz, CD_3OD) δ 165.1, 151.9, 151.5, 149.8, 137.6, 136.2, 132.5, 130.9, 119.7, 116.4, 112.9, 103.3, 88.8, 77.3, 74.3, 64.0, 45.7, 42.6, 13.0. **HR-ESI MS (*m/z*): [M+H]$^+$** calculated for $C_{21}H_{26}N_3O_4$ 384.1923, found 384.1945.

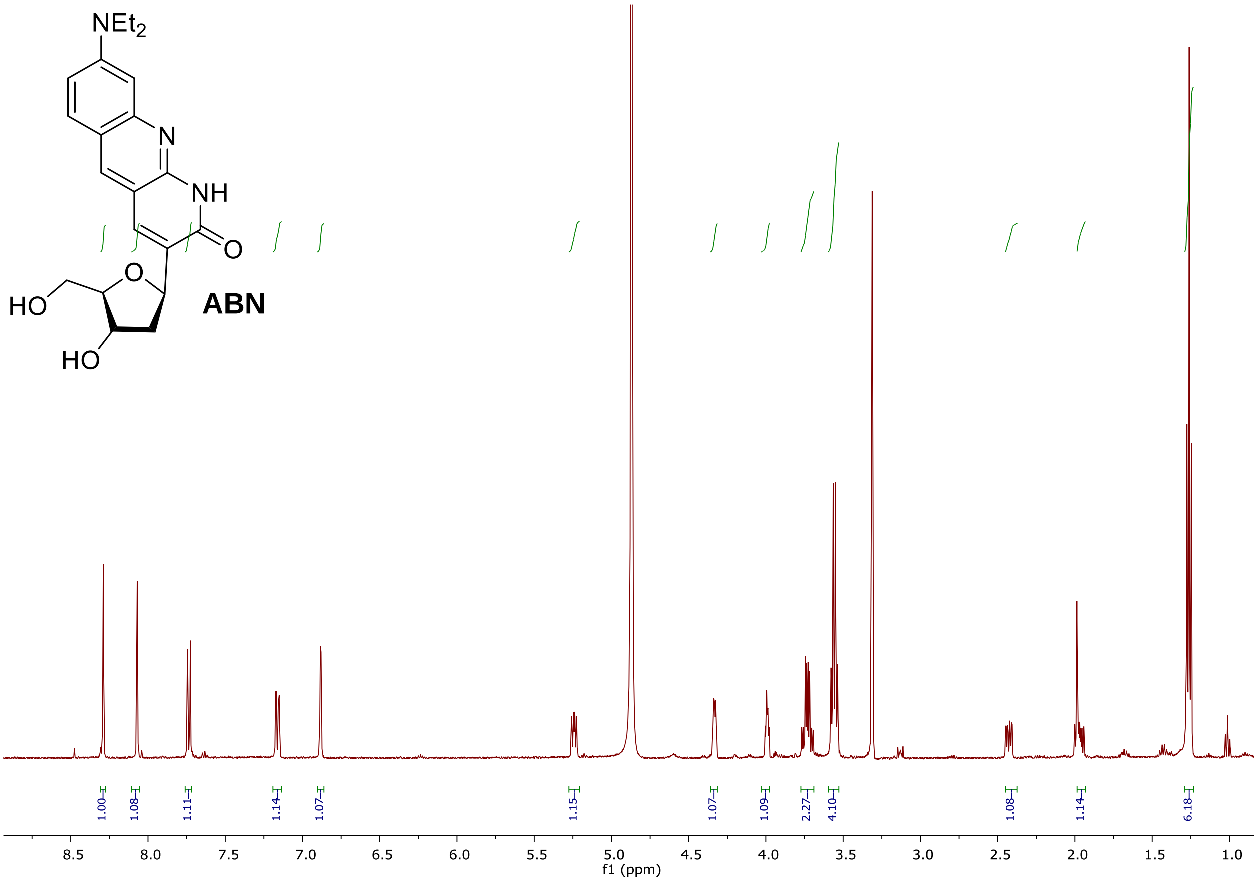

Fig. S1. **^{1}H NMR of** ABN (500 MHz; 298 K; CD_3OD).

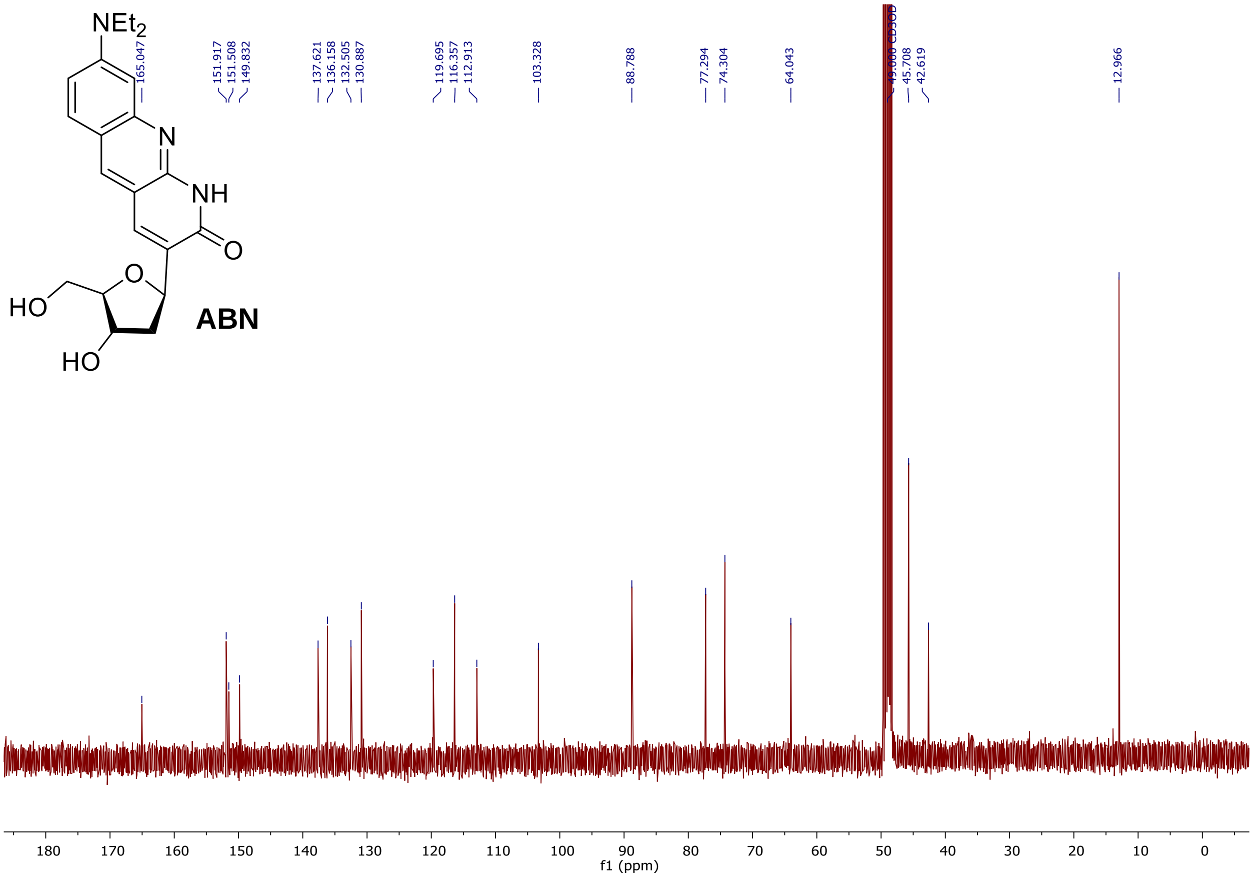

Fig. S2.^{13}C NMR of ABN (500 MHz; 298 K; CD_3OD).

1.3 Synthesis of oligonucleotides

ABN was prepared using the revised synthetic methods discussed above[1] and converted to the nucleoside phosphoramidite following the procedure that we have published previously. The ^{1}H and ^{13}C NMR spectra of the new phosphoramidite matched the spectra shown in this prior report. Oligonucleotides containing ABN were prepared by solid-phase DNA synthesis as performed by the W. M. Keck Foundation Oligonucleotide Synthesis Facility (Yale School of Medicine) using the ABN amidite. The HPLC-purified oligonucleotides were characterized by MALDI-TOF mass spectrometry and found to be consistent with calculated masses. Complementary DNA, LS02 and CS01 (with standard modifications of iSp9 and 3BioTEG) DNA sequences were purchased from Integrated DNA Technologies, Inc. (San Diego, CA).

1.4 Time-resolved fluorescence spectroscopy

Time-resolved fluorescence measurements

Lifetime measurements were carried out at 20 °C. Annealing of duplexes was carried out in the presence of an excess of the unlabelled complementary strand and hybridization was verified by UV absorption spectroscopy (hypochromism of the natural bases) before and after lifetime measurements to ensure the completeness of hybridization. Time-resolved fluorescence was measured using time-correlated single-photon counting on an Edinburgh Instruments spectrometer equipped with TCC900 photon counting electronics. A mode-locked Ti:sapphire laser (Coherent Mira pumped by Coherent Verdi), producing pulses of duration ~ 150 fs at a repetition rate of 76 MHz, was used as the excitation source. The pulse repetition rate was reduced to 4.75 MHz using a pulse picker (Coherent 9200) and the light was frequency-doubled using a Coherent 5-050 harmonic generator. Fluorescence response functions were recorded over 50 ns, in 4096 channels, and collected to a total of 10000 counts in the peak channel. Measurements were made at three emission wavelengths and the data were analysed globally, with lifetimes as the common parameters. Fluorescence response functions were fitted by iterative re-convolution, using Edinburgh Instruments software FAST.

In cases where emission occurred directly from the optically excited species, i.e. in the absence of excited-state tautomerism, the response function was fitted to a multi-exponential decay, equation S1.

$$I(t) = \sum_{i=1}^{n} A_i \exp\left(-\frac{t}{\tau_i}\right) \qquad \text{(S1)}$$

where *I* is the fluorescence intensity as a function of time, *t*, (normalised to the intensity at *t*=0); τ_i is the fluorescence lifetime of the *i*th decay component and A_i is the fractional amplitude (A-factor) of that component.

The average lifetime, $< \tau >$, of the emitting population is given by equation S2.

$$< \tau > = \frac{\sum_{i=1}^{n} A_i \tau_i}{\sum_{i=1}^{n} A_i} \qquad \text{(S2)}$$

The fraction of the steady-state emission intensity due to each species *i*, SS_i, is given by equation S3.

$$SS_i = \frac{A_i \tau_i}{\sum_{i=1}^{n} A_i \tau_i} \qquad \text{(S3)}$$

In cases where indirectly excited emission was observed, as a result of excited state tautomerism, manifested by the appearance of a rise time, the fitted function is given by equation S4. The observed response function consists of contributions from both directly-

excited emission from tautomer T1 and indirectly-excited emission from tautomer T2, as described in the kinetic analysis below.

$$\frac{I(t)}{I_{\max}} = B_{T1}\exp\left(-\frac{t}{\tau_1}\right) + B_{T2}\left(\exp\left(-\frac{t}{\tau_1}\right) - \exp\left(-\frac{t}{\tau_2}\right)\right) \qquad \text{(S4)}$$

where τ_1 and τ_2 are the fluorescence lifetimes of T1 and T2, respectively, and B_{T1} and B_{T2} are the amplitudes of the directly excited response function of T1 and the indirectly excited response function of T2, respectively.

Fractional contributions of T1 and T2 to steady state intensity are given by:

$$SS_{T1} = \frac{B_{T1}\tau_1}{B_{T1}\tau_1 + B_{T2}(\tau_1 - \tau_2)} \qquad \text{(S5)}$$

$$SS_{T2} = \frac{B_{T2}(\tau_1 - \tau_2)}{B_{T1}\tau_1 + B_{T2}(\tau_1 - \tau_2)} \qquad \text{(S6)}$$

Excited-state tautomerization kinetics and fluorescence response functions

A detailed discussion of the kinetics of excited-state intramolecular proton transfer has been reported by Woolfe et al.[2] Tautomer 1 (T1) is excited optically and undergoes tautomerization in the excited state (T1*) to form excited-state tautomer 2 (T2*).

$$\mathrm{T1} \xrightarrow{h\nu} \mathrm{T1}^* \xrightarrow{k_t} \mathrm{T2}^*$$

$\frac{-\mathrm{d}[\mathrm{T1}^*]}{\mathrm{d}t} = k_1[\mathrm{T1}^*]$

$\frac{-\mathrm{d}[\mathrm{T2}^*]}{\mathrm{d}t} = k_2[\mathrm{T2}^*] - k_t[\mathrm{T1}^*]$

where $k_1 = k_r^1 + k_{nr}^1 + k_t$ and $k_2 = k_r^2 + k_{nr}^2$

$[\mathrm{T1}^*] = [\mathrm{T1}^*]_0 \exp{(-k_1 t)}$

$[\mathrm{T2}^*] = \frac{k_t[\mathrm{T1}^*]_0}{k_2 - k_1}(\exp(-k_1 t) - \exp(-k_2 t))$

In terms of fluorescence lifetimes:

$[\mathrm{T1}^*] = [\mathrm{T1}^*]_0 \exp{(-\frac{t}{\tau_1})}$

$[\mathrm{T2}^*] = \frac{\tau_1 \tau_2 k_t [\mathrm{T1}^*]_0}{\tau_1 - \tau_2}\left(\exp\left(-\frac{t}{\tau_1}\right) - \exp\left(-\frac{t}{\tau_2}\right)\right)$

Where $\tau_1 = \frac{1}{k_1}$ and $\tau_2 = \frac{1}{k_2}$

Time-resolved fluorescence intensities (fluorescence response functions):

$I_{T1}(t) = k_r^1[\mathrm{T1}^*]_0 \exp\left(-\frac{t}{\tau_1}\right) = I_{T1}(0) \exp\left(-\frac{t}{\tau_1}\right)$

$I_{T2}(t) = \frac{k_r^2 \tau_1 \tau_2 k_t [\mathrm{T1}^*]_0}{\tau_1 - \tau_2}\left(\exp\left(-\frac{t}{\tau_1}\right) - \exp\left(-\frac{t}{\tau_2}\right)\right) = C\left(\exp\left(-\frac{t}{\tau_1}\right) - \exp\left(-\frac{t}{\tau_2}\right)\right)$

Note that because $\tau_1$is greater than τ_2 in the present case, τ_2 will appears as the rise time and τ_1 as the decay time for the T2 decay curve.

Since, for ABN, the emission spectra of T1 and T2 overlap, we see a sum of $I_{T1}(t)$ and $I_{T2}(t)$

$I(t) = \alpha I_{T1}(t) + \beta I_{T2}(t) = \alpha I_{T1}(0) \exp\left(-\frac{t}{\tau_1}\right) + \beta C\left(\exp\left(-\frac{t}{\tau_1}\right) - \exp\left(-\frac{t}{\tau_2}\right)\right)$

The observed response function, normalised to the maximum intensity is thus given by:

$\frac{I(t)}{\mathrm{I}_{\max}} = B_{T1} \exp\left(-\frac{t}{\tau_1}\right) + B_{T2}\left(\exp\left(-\frac{t}{\tau_1}\right) - \exp\left(-\frac{t}{\tau_2}\right)\right)$

where B_{T1} and B_{T2} are the amplitudes of the directly excited response function of T1 and the indirectly excited response function of T2, respectively.

1.5. Single-molecule total internal reflection fluorescence (smTIRF) microscopy

Single-molecule detection

Glass slides were etched with a piranha solution (3:1 sulfuric acid to hydrogen peroxide) for 10 minutes and were attached to the sticky-Slide VI 0.4 (Ibidi, Germany) flow chambers. The glass surface was coated with a solution containing 0.5 mg/ml poly-L-lysine/poly-ethylene glycol (PLL-PEG) and biotinylated PLL-PEG in a 20:1 ratio, followed by incubation for one hour. After this, the channels were washed with PBS, and 0.05 mg/ml streptavidin was added and incubated for 30 minutes. Excess streptavidin was removed by washing with PBS. Next, 100 nM of the capture strand, CS01, was introduced and allowed to incubate for 30 minutes. Unbound CS01 was subsequently washed away with PBS. Following this, 100 nM of the linker strand, LS01, was added. Any excess linker strand was washed away immediately before the introduction of GXC. To capture GXC, 10 nM of GXC was introduced and incubated for one minute. A control sample was prepared without the addition of streptavidin. To create an oxygen-depleted environment, oxygen scavenger GO/CAT-TX (consisting of 1 mg/ml glucose oxidase, 0.155 mg/ml catalase, 20 mM glucose, and 3 mM Trolox in PBS) was incubated for three minutes. Subsequently, the samples were examined using TIRF microscopy (Ti-2 inverted microscope, Nikon, Japan) and imaged using an EMCCD camera (iXon Life 897, Oxford Instruments). Florescence emission was collected by a 100X Apochromat TIRF oil objective (Nikon, Japan) and separated from excitation light using a dichroic mirror (zt488/640rpc-UF2, Chroma) and passed through two consecutive bandpass emission filters (ZET488/640m, ET535/70m, Chroma). The laser power at the objective was measured to be 2.1 mW. The brightness of ABN was analyzed using the imageJ Plugin TrackMate.[3] The ABN particles were localized using a difference of gaussian (DOG) detector with a radius of 3 pixels. The mean background intensity per pixel was calculated for each ABN particle. The intensity of a 3-pixel-radius region of interest (ROI), $I_{ROI_{in}}$, that enclose a single ABN particle, and that of a 5-pixel-radius ROI, $I_{ROI_{out}}$, that encloses ROI_{in} were measured. The average background intensity was calculated by:

$$\langle I_{bg} \rangle = \frac{I_{ROI_{out}} - I_{ROI_{in}}}{Area\ difference\ between\ ROI_{out}\ and\ ROI_{in}}$$

The net intensity of ROI_{in}, which corresponds to the net signal of a single ABN particle, was calculated by:

$$I_{net,ROI_{in}} = I_{ROI_{in}} - \left(\langle I_{bg} \rangle \times Area\ of\ ROI_{in}\right)$$

The net intensity per ABN particle was converted from counts to photons detected following the manufacturer's count conversion protocol. The distribution of photon counts was fitted using a log-normal distribution to obtain a mean photon number for the samples.

Single-molecule photodarkening kinetics

For the assessment of photodarkening kinetics, single-molecule trajectories were analyzed using the TrackMate plugin in ImageJ, employing the same particle detection method as in the brightness analysis. The fluorescence on-time distribution of the initial fluorescence event was obtained by plotting the probability distribution of the individual trajectory lengths. Surface-immobilized fluorescent molecules (F) transition to an unobservable state ($\emptyset$) at an observed rate constant, k_{obs}. This process occurs either through photodarkening (photobleaching/blinking) at a rate constant, k_{pho} (hereafter referred to simply as k), or through dissociation at a rate constant, k_{dis}. Both follow simple exponential kinetics.

$$\mathrm{F} \xrightarrow{k} \emptyset$$

$$\mathrm{F} \xrightarrow{k_{dis}} \emptyset$$

The probability distribution of molecules transitioning into the unobservable state is described by:

$$P(t) = \ k_{obs}e^{-k_{obs}} = (k + k_{dis})e^{-(k+k_{dis})}$$

The value of k_{dis} for ABN GXC(A) 10-mer and was determined in a separate experiment to be 0.010 (Fig. S10). Then k was calculated by:

$$k = k_{obs} - k_{dis}$$

No dissociation was observed for the Alexa488 12-mer within the experimental time frame.

2. Additional spectra, time-resolved fluorescence responses, and data tables for ABN in oligonucleotides

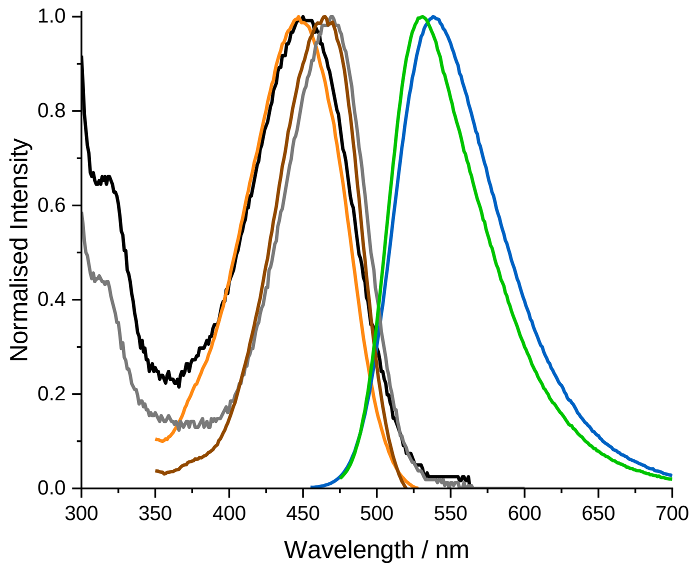

Fig. S3**.** Normalised absorption, excitation and emission spectra of ABN in duplex oligonucleotides GXC(A) and GXC(G). Spectra of GXC(A): absorption (black), excitation (orange) (recorded at emission wavelength 533 nm), emission (blue) (recorded at excitation wavelength 450 nm). Spectra of GXC(G): absorption (grey), excitation (brown) (recorded at emission wavelength 525 nm), emission (green) (recorded at excitation wavelength 470 nm).

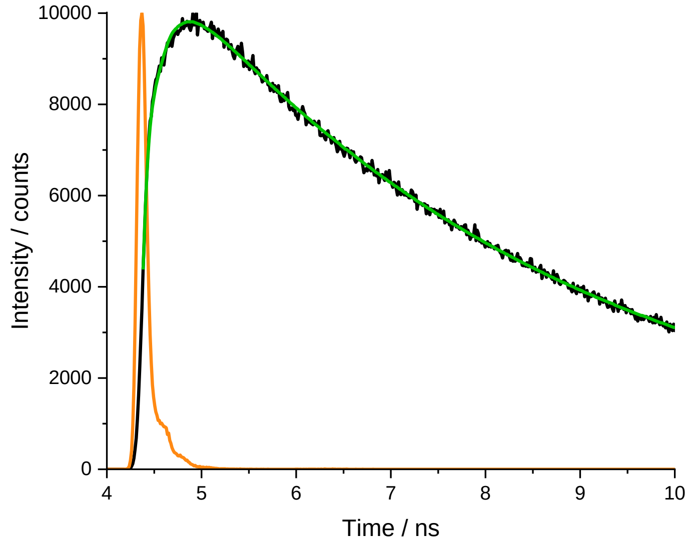

Fig. S4. Early-time portion of the fluorescence response function of ABN nucleoside in dioxane, showing the rise time. Experimental data (black), fitted function (green) and instrument response function) (orange).

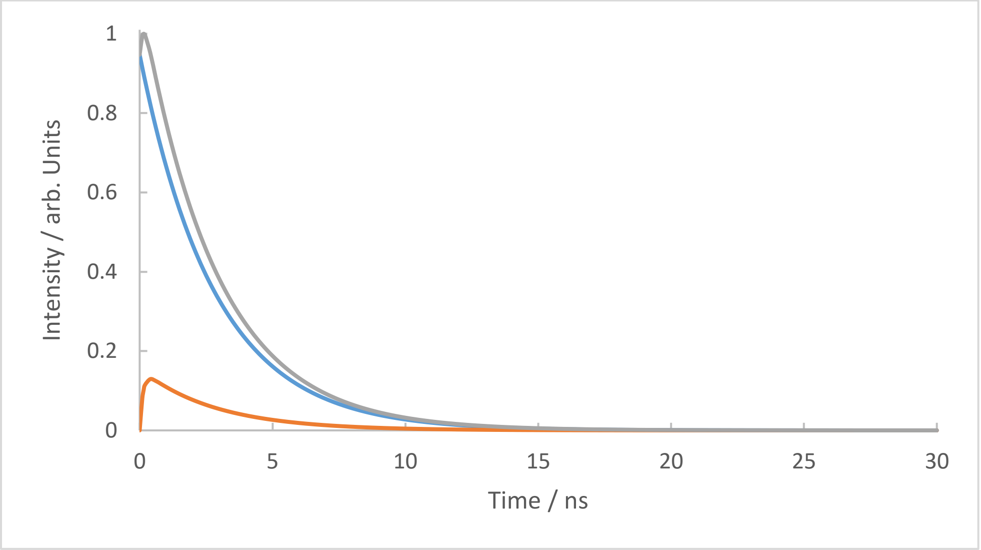

Fig. S5. The function fitted to the observed fluorescence response function of ABN in Tris buffer, excited at 440 nm and detected at 570 nm, is shown in grey. It consists of the sum of the response functions of T1 (blue) and T2 (orange). The emission of T1 (directly excited) shows a mono-exponential decay with a lifetime of 2.7 ns. The emission of T2 (populated by excited-state tautomerisation of T1) shows a rise time of 0.12 ns and a decay time of 2.7 ns.

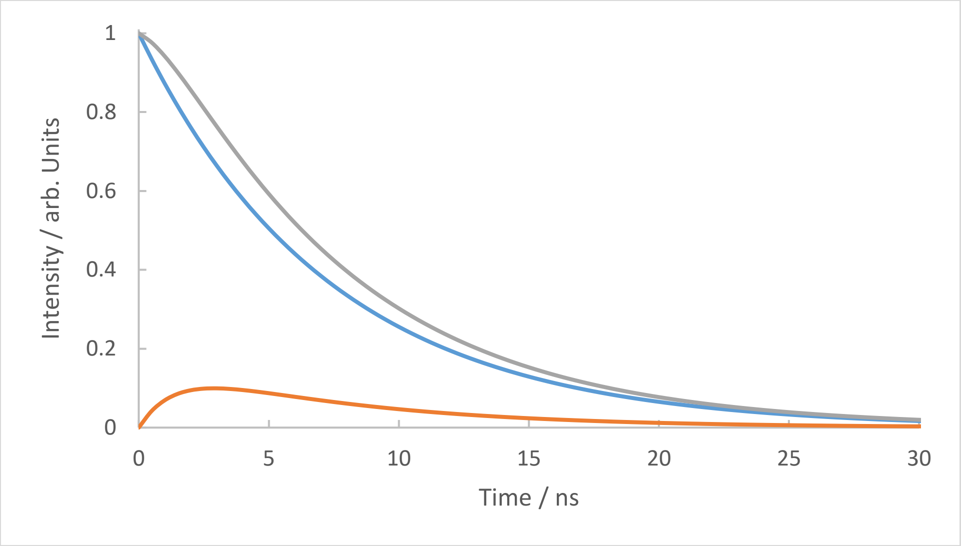

Fig. S6. The function fitted to the observed fluorescence response function of ABN in duplex oligonucleotide GXC(A) is shown in grey. It consists of the sum of the response functions of T1 (blue) and T2 (orange).

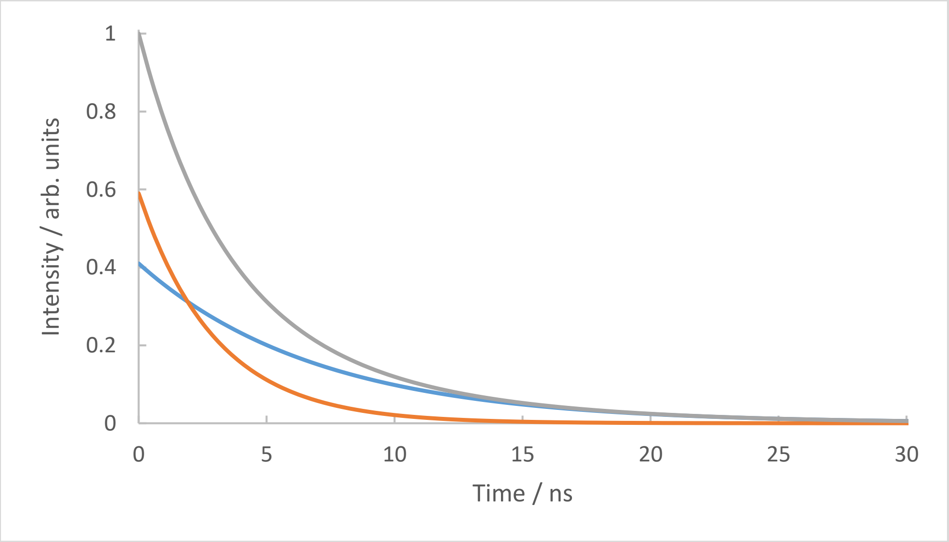

Fig. S7. The observed fluorescence response function (grey) of ABN in duplex oligonucleotide GXC(G) is shown in grey. It consists of the sum of the mono-exponential decays of T1 (blue) and T2 (orange).

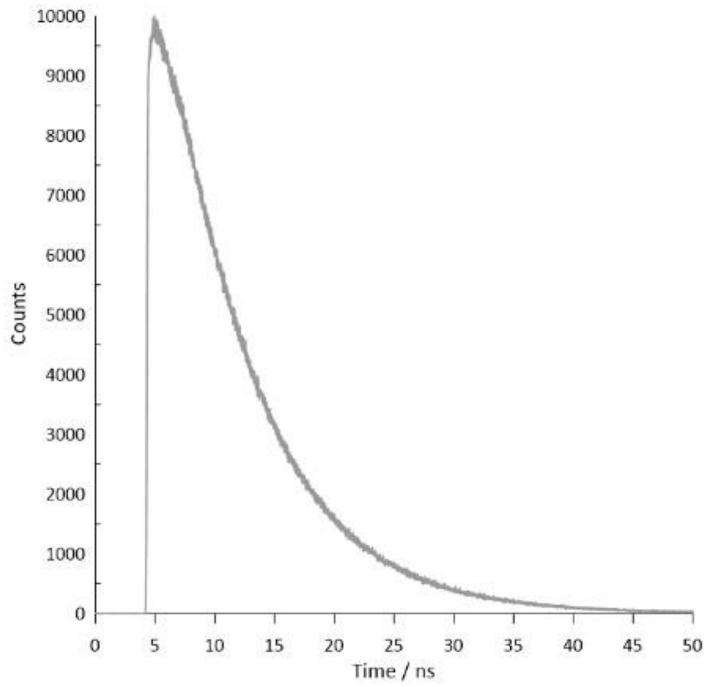

Fig S8 Experimentally observed fluorescence response function of AXA(A), recorded at an excitation wavelength of 440nm and an emission wavelength of 530 nm.

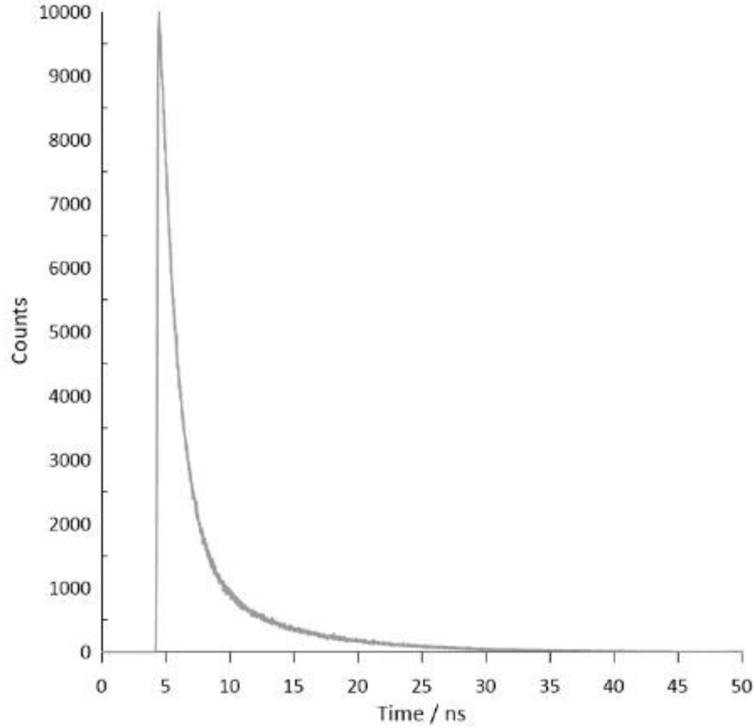

Fig. S9. Experimentally observed fluorescence response function of AXA(G), recorded at an excitation wavelength of 440nm and an emission wavelength of 520 nm.

Table S1. Fluorescence decay parameters for ABN in Tris buffer at an emission wavelength of 510 nm, with excitation at 440 nm.

τ_1 / ns	τ_2 / ns	τ_3 / ns		A_{T1}	A_{T2}	A_3
2.7	-	4.2		0.89		0.11

Table S2. Fluorescence decay parameters for ABN in duplex oligonucleotide AXA(A) at an emission wavelength of 500 nm, at the excitation wavelengths shown. The lifetimes are global across the emission wavelengths. The contribution of the T3 component to the steady-state intensity (%SS_3) is negligible.

λ_{ex}	τ_1 / ns	τ_2 / ns	τ_3 / ns	SS_1 / %	SS_2 / %	SS_3 / %
420				77	22	1
440	7.2	1.8*	0.34	78	21	1
460				80	19	1

Table S3. Fluorescence decay parameters for ABN in duplex oligonucleotide AXA(G) at an emission wavelength of 490 nm, at the excitation wavelengths shown. The lifetimes are global across the emission wavelengths. The contribution of the T3 component to the steady-state intensity (%SS_3) is very small.

λ_{ex} / nm	τ_1 / ns	τ_2 / ns	τ_3 / ns	A_1	A_2	A_3	%SS_3
420				0.10	0.55	0.35	6.0
440	7.0	1.4	0.27	0.07	0.61	0.33	6.2
460				0.05	0.61	0.34	6.9

Table S4. Fluorescence response parameters of the tautomers of ABN in duplex oligonucleotide AXA(A), at the excitation and emission wavelengths shown; * indicates that this lifetime component was observed as a rise-time. Lifetimes were obtained by global analysis of response functions recorded at all excitation and emission wavelengths.

λ_{ex} / nm	λ_{em} / nm	τ_1 / ns	τ_2 / ns	B_{T1}	B_{T2}	$\%SS_{T1}$	$\%SS_{T2}$
420	500[a]	7.2	1.8*	1.0	0.38	77	22
	530			0.99	0.41	76	23
	560			0.98	0.47	73	27
440	500[a]			1.0	0.36	78	21
	530			0.99	0.40	77	23
	560			0.99	0.44	75	25
460	500[a]			1.0	0.32	80	19
	530			1.0	0.34	79	21
	560			1.0	0.37	78	22

[a] A minor additional decay component was present at this emission wavelength, see Table S2.

Table S5. Fluorescence decay parameters of the tautomers of ABN in duplex oligonucleotide AXA(G). Lifetimes (τ_i) were obtained by global analysis of response functions recorded over all excitation and emission wavelengths. Fractional amplitudes (A_i) and % contributions to the steady-state intensity (%SS_i) depend on both excitation and emission wavelength. [a] A minor additional decay component was present at this emission wavelength (see Table S3).

λ_{ex} / nm	λ_{em} / nm	τ_1 / ns	τ_2 / ns	A_1	A_2	%SS_1	%SS_2
	490 [a]			0.10	0.55	43	51
420	520			0.21	0.79	56	44
	550			0.37	0.63	73	27
	490 [a]			0.07	0.60	33	61
440	520	7.0	1.4	0.14	0.86	45	55
	550			0.26	0.74	63	37
	490 [a]			0.05	0.61	26	67
460	520			0.12	0.88	39	61
	550			0.22	0.78	57	43

Table S6. The number-average lifetime of the emission of ABN in duplex oligonucleotide AXA(G), as a function of excitation and emission wavelength.

λ_{ex} / nm	λ_{em} / nm	$\langle\tau\rangle$ / ns
	490	1.6
420	520	2.6
	550	3.5
	490	1.4
440	520	2.2
	550	2.9
	490	1.3
460	520	2.1
	550	2.6

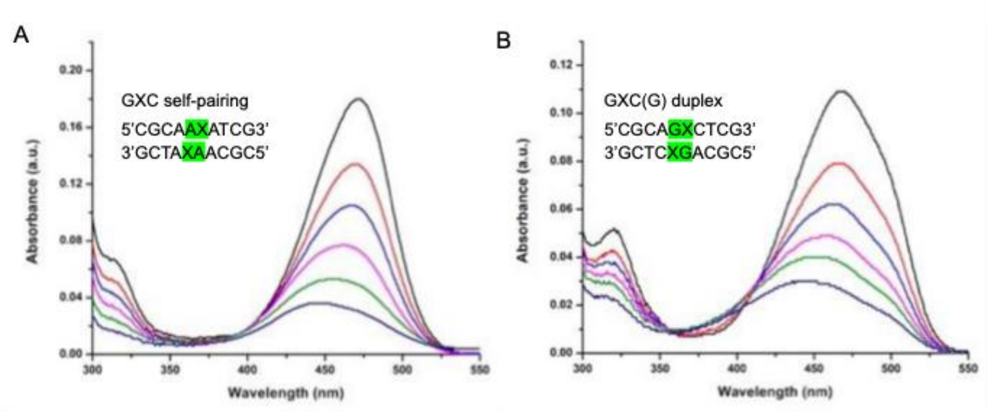

FigS10. Concentration dependency of the absorption spectra of GXC in single-strand and duplex forms. (A) The absorption maximum of double-stranded GXC(G) shifts from ~450 nm to ~470 nm as the concentration increases. This gradual red shift is explained by a change in the relative populations of the T-like T1 (which absorbs at shorter wavelengths) and the C-like T2 (which absorbs at longer wavelengths). At low concentrations, the duplexes tend to dissociate, increasing the relative population of the unpaired T1. At higher concentrations, base pairing with G increases the relative population of T2. (B) The concentration-dependent red-shift of single-stranded GXC is accounted for an increase in the T2 population due to self-pairing. Reprinted from Ref (1).

3. Supplementary figures and table for smTIRF

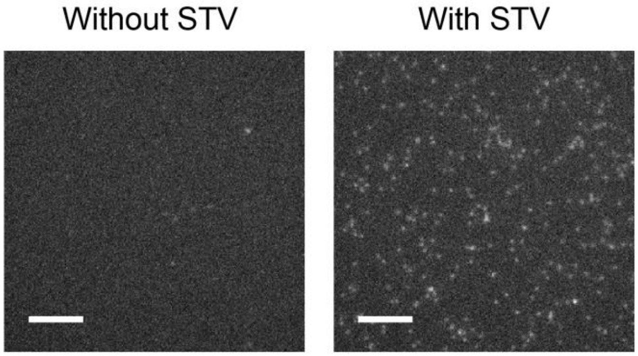

Fig. S11. Selective immobilization of ABN to a glass surface. ABN-containing GXC molecules were captured to streptavidin-coated glass surface through DNA hybridization with a complementary linker strand displayed by a biotinylated capture strand. Minimal to no GXC immobilization occurred in the absence of streptavidin. Scale bars represent 5 μm.

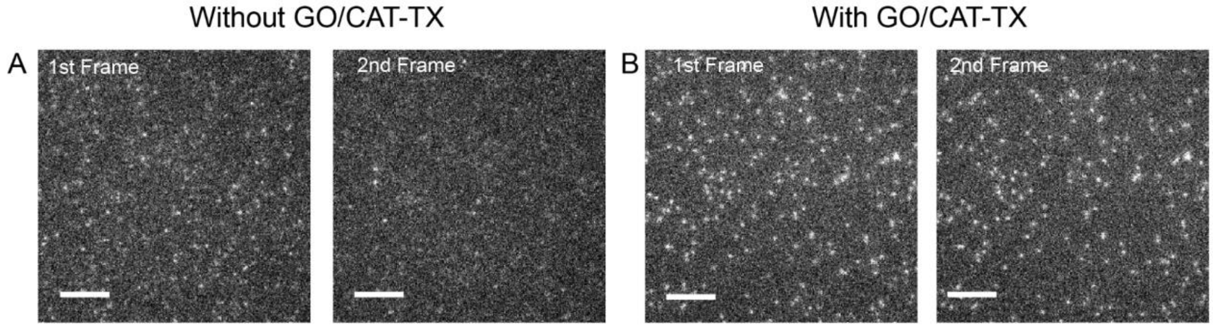

Fig. S12. Effects of GO/CAT-TX on ABN photobleaching. (A) Without GO/CAT-TX, significant ABN photobleaching occurred in the second frame with a 100 ms exposure time. (B) The addition of GO/CAT-TX improved the photostability of ABN, with most ABN surviving to the second frame. Scale bars represent 5 μm.

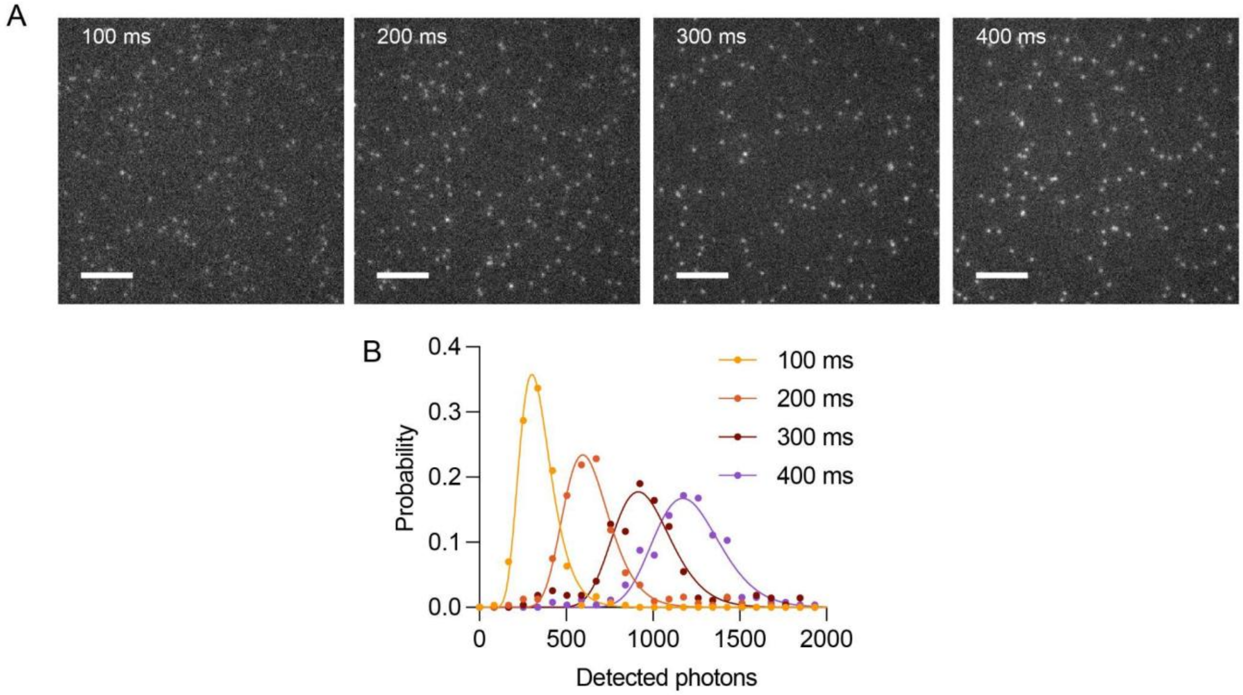

Fig. S13. (A) ABN measurements at different exposure times ranging from 100 ms to 400 ms and (B) their corresponding photon distributions.

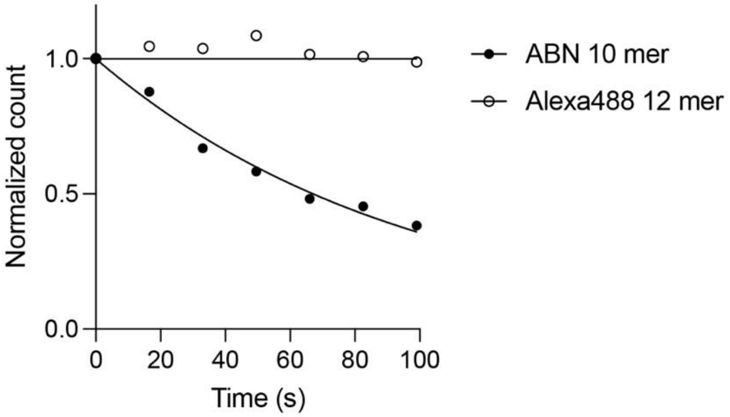

Fig. S14. The dissociation kinetics of ABN (10-mer) and Alexa488 (12-mer) were analyzed by counting the number of bound molecules over time. To minimize the effects of photobleaching, a new, unexposed area was imaged by moving the sample 150 µm between measurements. The dissociation rate constant, k_{dis}, for ABN was estimated to be 0.010 by fitting a single exponential equation: $y = e^{-(k_{dis}t)}$. No detectable dissociation was observed for Alexa488 up to 100 seconds. At time zero, the number of molecules (*N*) was approximately 700 for ABN (10-

mer) and 400 for Alexa488 (12-mer). Sequence of Alexa488 (12-mer): /5AlexF488N/CCGCAATATC GC. Corresponding linker: TTGAA AGATCACACATTCATGTT TTGGGCGATATTGCGG.

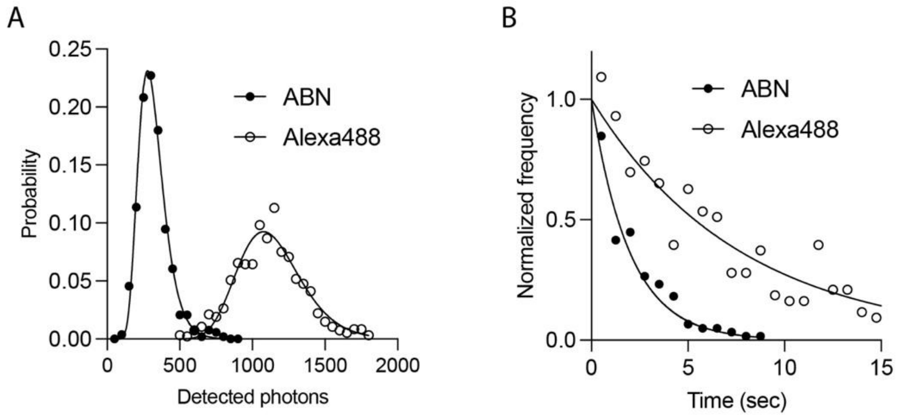

Fig. S15. Comparison of ABN and Alexa488 in the presence of GO/CAT-TX. (A) Histogram showing the number of detected photons at a 100 ms exposure. The mean photon number was estimated to be 289 ± 187 (N = 744) for ABN and 1141 ± 705 (N = 1064) for Alexa488. (B) Plot of photobleaching/blinking kinetics of ABN and Alexa488. The photodarkening rate constant, *k*, was estimated to be 0.13 s^{-1} for Alexa488 and 0.50 s^{-1} for ABN.

Table S7. A comparison of ABN's and Alexa488's properties as fluorophores in smTIRF of oligonucleotides.

Property	ABN	Alexa488
$\lambda_{abs,max}$	450 nm[a]	495 nm[b]
$\lambda_{em,max}$	538 nm[a]	519 nm[b]
ε at λ_{max}	20,000 $M^{-1}cm^{-1}$ [c]	73,000 $M^{-1}cm^{-1}$ [b]
Φ_{em}	0.48 [d]	0.92 [b]
mean photon number[e,f]	289 ± 187	1141 ± 705
photodarkening rate constant k[e,f]	0.50 s^{-1}	0.13 s^{-1}

[a] Data from the GXC(A) hybrid duplex. [b] Data are widely used reference values for Alexa488 in PBS, as provided by the manufacturer. [c] The value presented is for the free ABN nucleoside in 1× PBS buffer at pH 7.4. [d] Data are presented for the GXC(A) hybrid duplex. [e] smTIRF data for ABN are presented for GXC immobilized as shown in Fig 2 and discussed above. [f] smTIRF data for Alexa488 are presented for the Alexa488 12-mer as discussed in Fig. S14 and discussed above.

4. References

(1) Samaan, G. N.; Wyllie, M. K.; Cizmic, J. M.; Needham, L.-M.; Nobis, D.; Ngo, K.; Andersen, S.; Magennis, S. W.; Lee, S. F.; Purse, B. W. *Chem. Sci.* **2021**, *12*, 2623.
(2) Woolfe, G. J.; Thistlethwaite, P. J. *J. Am. Chem. Soc.* **1981**, *103*, 6916.
(3) Tinevez, J.-Y.; Perry, N.; Schindelin, J.; Hoopes, G. M.; Reynolds, G. D.; Laplantine, E.; Bednarek, S. Y.; Shorte, S. L.; Eliceiri, K. W. *Methods* **2017**, *115*, 80.
